# Sensitivity analysis of a strongly-coupled human-based electromechanical cardiac model: Effect of mechanical parameters on physiologically relevant biomarkers

**DOI:** 10.1016/j.cma.2019.112762

**Published:** 2020-04-01

**Authors:** F. Levrero-Florencio, F. Margara, E. Zacur, A. Bueno-Orovio, Z.J. Wang, A. Santiago, J. Aguado-Sierra, G. Houzeaux, V. Grau, D. Kay, M. Vázquez, R. Ruiz-Baier, B. Rodriguez

**Affiliations:** a Department of Computer Science, University of Oxford, Oxford OX1 3QD, United Kingdom; b Department of Engineering Science, University of Oxford, Oxford OX3 7DQ, United Kingdom; c Barcelona Supercomputing Center – Centro Nacional de Supercomputación, Barcelona 08034, Spain; d ELEM Biotech, Spain; e Mathematical Institute, University of Oxford, Oxford OX2 6GG, United Kingdom; f Universidad Adventista de Chile, Casilla 7-D, Chillan, Chile

**Keywords:** Cardiac electromechanics, Sensitivity analysis, Multiscale simulations, Finite element method, High-performance computing

## Abstract

The human heart beats as a result of multiscale nonlinear dynamics coupling subcellular to whole organ processes, achieving electrophysiologically-driven mechanical contraction. Computational cardiac modelling and simulation have achieved a great degree of maturity, both in terms of mathematical models of underlying biophysical processes and the development of simulation software.

In this study, we present the detailed description of a human-based physiologically-based, and fully-coupled ventricular electromechanical modelling and simulation framework, and a sensitivity analysis focused on its mechanical properties. The biophysical detail of the model, from ionic to whole-organ, is crucial to enable future simulations of disease and drug action. Key novelties include the coupling of state-of-the-art human-based electrophysiology membrane kinetics, excitation–contraction and active contraction models, and the incorporation of a pre-stress model to allow for pre-stressing and pre-loading the ventricles in a dynamical regime. Through high performance computing simulations, we demonstrate that 50% to 200%−1000% variations in key parameters result in changes in clinically-relevant mechanical biomarkers ranging from diseased to healthy values in clinical studies. Furthermore mechanical biomarkers are primarily affected by only one or two parameters. Specifically, ejection fraction is dominated by the scaling parameter of the active tension model and its scaling parameter in the normal direction ($k_{\text{ort}\;2}$); the end systolic pressure is dominated by the pressure at which the ejection phase is triggered ($P_{\text{ej}}$) and the compliance of the Windkessel fluid model (C); and the longitudinal fractional shortening is dominated by the fibre angle (ϕ) and $k_{\text{ort}\;2}$. The wall thickening does not seem to be clearly dominated by any of the considered input parameters.

In summary, this study presents in detail the description and implementation of a human-based coupled electromechanical modelling and simulation framework, and a high performance computing study on the sensitivity of mechanical biomarkers to key model parameters. The tools and knowledge generated enable future investigations into disease and drug action on human ventricles.

## Highlights

• Detailed description of human fully-coupled ventricular electromechanical model.
• Sensitivity analysis reveals parameter variations effects on mechanical biomarkers.
• Simulations yield biomarker values ranging from healthy to diseased conditions.
• Each biomarkers is primarily affected by one or two of the parameters tested.
• Tools and knowledge generated enable investigations into disease and drug action.

## 1 Introduction

**Scope.** Cardiac disease is the leading cause of death worldwide, affecting millions of people every year [1]. Disease generally affects the human heart through complex mechanisms, involving from subcellular processes such as ionic currents, to whole-organ properties such as tissue structure; and impairing, in one way or another, the heart’s primary role of pumping blood through mechanical contraction and relaxation driven by electrophysiological activity. The great complexity of the pathophysiological mechanisms involved in cardiac electromechanical function is difficult to assess in clinical and experimental settings, due to ethical and practical limitations, and also limited access to human tissue. Novel human-based approaches are therefore required to accelerate very much needed improvements in diagnosis and treatment [2].

Computational multiscale modelling and simulations provide a powerful framework to dissect the highly nonlinear processes involved in cardiac electromechanical disease. In cardiac science, computational modelling and simulation has been an active area of research, at the forefront of computational biomedicine [3]. For the last five decades, cell electrophysiology, excitation–contraction coupling and active tension models have been developed in close iteration with experiments. Detailed models and simulations of human ventricular electrophysiology have replicated experimental and clinical recordings in a range of healthy and disease conditions, and also under drug action (see for instance [3], [4], [5]). Their maturity has triggered interest and impact beyond academia, such as the adoption of the *state-of-the-art* O’Hara–Rudy (ORd) model [6] for industrial and regulatory purposes, within the CiPA initiative sponsored by the US Food and Drug Administration [7]. Studies have shown prediction of drug-induced clinical arrhythmic risk in human with 89% accuracy while data obtained from previously conducted animal studies for similar datasets showed up to 75% accuracy [8]. Furthermore, recently a new model of human-based ventricular active contraction was proposed and evaluated by Land et al. [9] using experimental recordings obtained in human tissue preparations. However, the coupling and incorporation of these two models in the framework of organ-level ventricular simulations has not been achieved yet.

Progress has also been impressive in enabling simulations considering multi-physics coupling including electrophysiology and mechanics for whole heart dynamics (see for instance [10], [11], [12], [13], [14], [15], amongst others). Some of these studies have also considered fully coupled electro-mechanico-fluidic ventricular dynamics, where an explicit representation of the fluid via the Navier–Stokes equations is considered [13], [14], [16]. Building on previous work mainly from [14], [17], [18], [19], [20], in this study we describe a human-based physiologically-detailed, and fully-coupled ventricular electromechanical model together with a suitable simulation framework and a sensitivity analysis focusing on its mechanical properties. One of the novelties of this formulation is the level of detail considered (from ionic to whole-organ), which is crucial to eventually enable simulations of disease and drug action. Other key additions include coupled *state-of-the-art* human-based electrophysiology membrane kinetics, excitation–contraction and active contraction models [9], [21], and the implementation of a dedicated model that allows us to consider pre-stressing and pre-loading of the ventricles in a dynamical regime. This overcomes some of the limitations associated with one or more of the following approaches: quasi-static instead of dynamic formulations (i.e. ignoring inertial effects, which may indeed be relevant in cardiac electromechanics [22], [23]), simplified models for cell electrophysiology and/or active tension [24], [25], the lack of pre-stress and/or lack of representation of the four phases of the cardiac cycle [18], [26], and lack of an appropriate representation of epicardial mechanical boundary conditions [27], [28]. Moreover, to enable the reproducibility of the results, we also include a full description of the numerical schemes and their computational implementation, giving sufficient detail for each model component.

Furthermore, through high performance computing (HPC), we conduct a sensitivity analysis in which we quantify variations in simulated mechanical biomarkers caused by varying key parameters in the human ventricular electromechanical model. Whereas previous studies have investigated the variability generated by electrophysiological parameters [29], [30], the role of variability in mechanical parameters on mechanical biomarkers in the human heart has not been systematically assessed yet. After recalling classical approaches to deal with uncertainty in parameter values, we perform HPC simulations varying key mechanical parameters within appropriate ranges, we then quantify physiologically-relevant biomarkers, and we finally validate our results against clinically-relevant values.

Notation The mathematical notation defined in this section largely follows the one used in [13], [31], adopting tensor notation throughout this study. Unless otherwise specified, scalars are denoted with Greek or Latin italic characters (e.g. α or a, respectively); vectors, or first-order tensors, are denoted by Latin bold lower-case characters (e.g. a); second-order tensors are denoted with Greek or Latin bold upper-case characters (e.g. σ or A, respectively); and fourth-order tensors are denoted by Latin double-barred upper-case characters (e.g. A). Reference and spatial coordinates are respectively indicated with X and x. Reference and spatial configurations of a body are respectively denoted with $\Omega_{0}$ and Ω, with $\partial \Omega_{0}$ and ∂Ω being their corresponding boundaries. Subscripts are used for naming purposes and, when relevant, they are followed, after a space, by a time step, iteration number, or additional naming subscripts; superscripts are also used for naming purposes. Additional notation details are given in Appendix A.

## 2 Materials and methods

### 2.1 Geometry of the left ventricular model

A simplified left ventricular geometry is modelled as an ellipsoid, truncated at the base. The geometry was obtained from the cardiac mechanics benchmark in [32], and isotropically scaled to obtain an end-diastolic volume (EDV) similar to that of the human left ventricle (EDV = 159.43 ml) [33]. The finite element (FE) mesh is composed of linear tetrahedra, and has a maximum edge length of ∼0.025 cm, resulting in ∼33M elements and ∼5.6M nodes; the used step size ranges from 0.001 to 0.01 ms. Such mesh size is needed for the convergence of numerical algorithms as discussed below.

### 2.2 Description of the governing equations

**Fig. 2.1:**
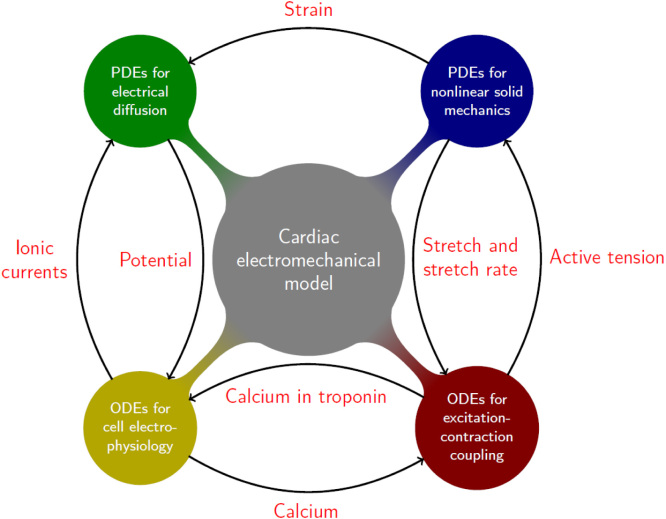
Diagram of the human ventricular electromechanical modelling and simulation framework, which includes: **(blue)** nonlinear solid mechanics, **(green)** electrical propagation, **(yellow)** ventricular cell electrophysiology, and **(red)** active-tension plus excitation-contraction. The arrows represent the different coupling mechanisms and their corresponding directions, with the quantities written in red being exchanged between the different components of the model. (For interpretation of the references to colour in this figure legend, the reader is referred to the web version of this article.)

The main components of the human-based ventricular electromechanical model are: (1) the anisotropic description of the mechanical behaviour of ventricular muscle (Fig. 2.1 Blue “blob”), (2) the electrical propagation model (Fig. 2.1 Green “blob”), (3) the cell electrophysiology model including calcium dynamics (Fig. 2.1 Yellow “blob”), and (4) the active-tension model coupled to the excitation–contraction model (Fig. 2.1 Red “blob”). As in [13], the present subsection describes physiological models and coupling mechanisms for each of these phenomena, primarily focusing on their mathematical structure. A complete description of the specific way of coupling human-based electrophysiology and active tension models is one of the novelties of this study, and the results are detailed and thoroughly discussed in Section ??.

#### 2.2.1 Ventricular mechanical activity

**Fig. 2.2:**
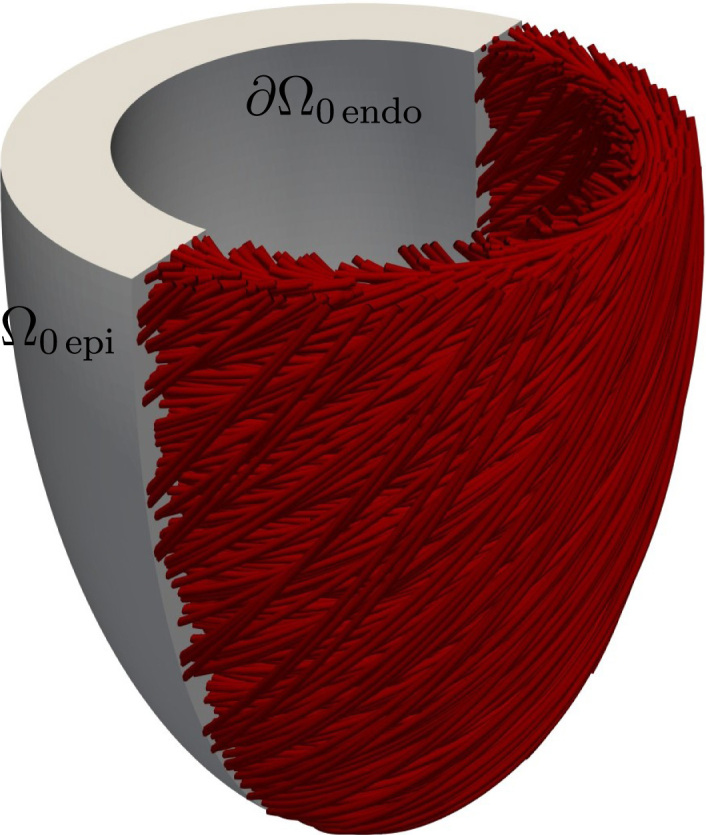
Left ventricular geometry, including local fibre architecture and domain boundaries. The centreline of the ventricular cavity is aligned with the $x_{3}$-axis.

The end-diastolic configuration of the left ventricle is considered as the region $\Omega_{0}\subset R^{3}$ at t=0, also referred to as *reference*, or *material*, configuration, with the following boundaries: epicardium $\partial \Omega_{0\;\text{epi}}$ and left ventricular endocardium $\partial \Omega_{0\;\text{endo}}$ (Fig. 2.2). The motion of the ventricles is defined through the deformation operator $\phi :\Omega_{0}\times R\to R^{3}$ which maps the position of every location in the ventricles $\Omega_{0}$ onto the *spatial*, or *current*, configuration $\Omega =\phi \left(\Omega_{0},t\right)$ for a time t∈(0,T]. The deformation gradient tensor field F is defined as $F=\nabla_{X}\phi$, and maps tangent vectors from the reference configuration onto the spatial configuration.

The mechanical behaviour of ventricular tissue is characterised by the balance of linear momentum in a total Lagrangian formulation, considering inertial contributions, but ignoring volumetric forces (e.g. gravitational effects or body loads), such that

(2.1a) $$\rho_{0}\frac{\partial^{2}u}{\partial t^{2}}-\nabla_{X}\cdot \left(FS\right)=0,\;\text{in}\;\Omega_{0}\times \left(0,T\right],$$

(2.1b) $$FSn_{0}-JP_{\text{endo}}F^{-\text{T}}n_{0}=0,\;\text{on}\;\partial \Omega_{0\;\text{endo}}\times \left(0,T\right],$$

(2.1c) $$FSn_{0}-\left(k_{\text{epi}}u\cdot n_{0}\right)n_{0}=0,\;\text{on}\;\partial \Omega_{0\;\text{epi}}\times \left(0,T\right],$$

where $\rho_{0}$ is the (initial) density of the considered material in the reference configuration, u is the field of displacements, F is the deformation gradient $F=I+\nabla_{X}u$, S is the second Piola–Kirchhoff stress, $P_{\text{endo}}$ is the pressure applied to the endocardial surface and obtained through the Windkessel model described below, $k_{\text{epi}}$ is the stiffness corresponding to the elastic spring boundary condition applied to the epicardium, and $n_{0}$ is the outward normal defined on the whole material boundary $\partial \Omega_{0}$. Note that in Eq. (2.1c) $\left(k_{\text{epi}}u\cdot n_{0}\right)n_{0}\equiv An_{0}$, where A could be seen as a linear operator which projects u to the subspace spanned by $n_{0}$ and then scales it by $k_{\text{epi}}$; this would act as a spring which is positioned normally to the epicardial surface $\partial \Omega_{0\;\text{epi}}$, and with constant stiffness $k_{\text{epi}}$.

The considered constitutive law for the passive mechanical behaviour of ventricular tissue is a nearly, or quasi-, incompressible version of Holzapfel and Ogden [34]; justified by *in-vivo* measurements of the material properties of the myocardium [35], [36], it is assumed that the ventricular tissue admits a small degree of compressibility. The strain energy density function defining the orthotropic and hyperelastic continuum is

$$\psi \left(C\right)=\psi_{\text{vol}}\left(J\right)+{\bar{\psi }}_{\text{iso}}\left(\bar{C}\right)+{\bar{\psi }}_{\text{aniso}}\left(\bar{C},f_{0},s_{0}\right),$$

where its volumetric, and isochoric isotropic/anisotropic contributions are respectively defined as

$$\psi_{\text{vol}}\left(J\right)=\frac{K}{2}{\left(J-1\right)}^{2},\;{\bar{\psi }}_{\text{iso}}\left(\bar{C}\right)=\frac{a}{2b}\left({\text{e}}^{b\left({\bar{I}}_{1}-3\right)}-1\right),$$

$${\bar{\psi }}_{\text{aniso}}\left(\bar{C},f_{0},s_{0}\right)=\overset{2}{\underset{i=\text{f},\text{s}}{\sum }}\frac{a_{i}}{2b_{i}}\left({\text{e}}^{b_{i}{\left({\bar{I}}_{4i}-1\right)}_{+}^{2}}-1\right)+\frac{a_{\text{fs}}}{2b_{\text{fs}}}\left({\text{e}}^{b_{\text{fs}}{\bar{I}}_{\text{8fs}}^{2}}-1\right),$$

and J=detF, $C=F^{\text{T}}F$, $\bar{x}=J^{-\frac{2}{3}}x$ and $x_{+}=\frac{1}{2}\left(x+\left|x\right|\right)$; $f_{0}$ and $s_{0}$ are the fibre and sheet directions in the reference configuration. These, together with the normal direction in the reference configuration ${\bar{n}}_{0}$ form a right-handed orthonormal set of basis vectors (note the over-bar, which emphasises the difference between the normal to the fibre-sheet plane and the normal to a boundary of the considered domain, as previously specified). The parameters $K,a,a_{i},a_{\text{fs}}$ have stress dimensions, whereas $b,b_{i},b_{\text{fs}}$ are dimensionless parameters; all considered positive, and K being the bulk modulus. The term $I_{i}$ is the ith invariant of the right Cauchy–Green strain tensor C, defined as

$$I_{1}=\text{tr}\;C,\;I_{4\text{f}}=f_{0}\cdot Cf_{0},\;I_{4\text{s}}=s_{0}\cdot Cs_{0},\;I_{8\text{fs}}=\frac{1}{2}\left(f_{0}\cdot Cs_{0}+s_{0}\cdot Cf_{0}\right).$$

From these specifications, the precise form of the passive contribution to the second Piola–Kirchhoff stress tensor can be obtained as

$$S_{\text{pas}}=2\frac{\partial \psi }{\partial C}=K\left(J-1\right)JC^{-1}+J^{-\frac{2}{3}}a\;{\text{e}}^{b\left({\bar{I}}_{1}-3\right)}\left(I-\frac{1}{3}I_{1}C^{-1}\right)\;+2J^{-\frac{2}{3}}a_{\text{f}}{\left({\bar{I}}_{\text{4f}}-1\right)}_{+}{\text{e}}^{b_{\text{f}}{\left({\bar{I}}_{\text{4f}}-1\right)}_{+}^{2}}\left(f_{0}⊗f_{0}-\frac{1}{3}I_{\text{4f}}C^{-1}\right)\;+2J^{-\frac{2}{3}}a_{\text{s}}{\left({\bar{I}}_{\text{4s}}-1\right)}_{+}{\text{e}}^{b_{\text{s}}{\left({\bar{I}}_{\text{4s}}-1\right)}_{+}^{2}}\left(s_{0}⊗s_{0}-\frac{1}{3}I_{\text{4s}}C^{-1}\right)\;+2J^{-\frac{2}{3}}a_{\text{fs}}{\bar{I}}_{\text{8fs}}\;{\text{e}}^{b_{\text{fs}}{\bar{I}}_{\text{8fs}}^{2}}\left[\frac{1}{2}\left(f_{0}⊗s_{0}+s_{0}⊗f_{0}\right)-\frac{1}{3}I_{\text{8fs}}C^{-1}\right].$$

Equation (2.1b) describes the effect of blood flow on the left ventricle. This was implemented through a state machine with four (five counting the initiation) phases, one for each of the cardiac phases during a heartbeat: isovolumetric contraction, ejection, isovolumetric relaxation, and filling; specified as follows. For additional details on the Windkessel model, see e.g. [24].

• *Initiation.* This phase is needed in order to bring the system to the end of diastole. The ventricular pressure applied to the endocardium is brought up to healthy left ventricular end-diastolic pressure linearly throughout the phase. In order to keep the volume constant during this phase, a pre-stress $\sigma_{0}$ is assumed to follow the fibre direction and is initialised at t=0 with a relatively small value and is further adjusted during this phase via the relation
  (2.2) $$\text{d}\sigma_{0}=\frac{1}{C_{\text{p}\;0}}\text{d}V_{\text{endo}}+\frac{1}{C_{\text{v}\;0}}\frac{\text{d}V_{\text{endo}}}{\text{d}t},$$
  where $C_{\text{p}\;0}$ and $C_{\text{v}\;0}$ can be considered as the inverse of penalty terms for volume difference and volume rate, respectively. The last summand is used as a stabilisation term in order to avoid oscillations of the ventricular pressure due to inertial effects. The pre-stress $\sigma_{0}$ is kept constant for the rest of simulation, at the converged value obtained in the last time step of this phase.
• *Isovolumetric contraction.* At this phase the aortic valve is closed, and the ventricular pressure increases due to contraction of the ventricles caused by electrical activation, while keeping the volume constant. The ventricular pressure required to maintain the condition of constant ventricular volume is obtained via
  (2.3) $$\text{d}P_{\text{endo}}=-\frac{1}{C_{\text{p}}}\text{d}V_{\text{endo}}-\frac{1}{C_{\text{v}}}\frac{\text{d}V_{\text{endo}}}{\text{d}t},$$
  where $C_{\text{p}}$ and $C_{\text{v}}$ can be considered as the inverse of penalty terms for volume difference and volume rate, respectively. Again, the last summand is used as stabilisation against spurious oscillations of the ventricular pressure, which might occur due to inertial effects.
• *Ejection.* When the ventricular pressure surpasses the arterial pressure, the aortic valve opens and the ejection phase begins, eventually leading to a reduction in ventricular volume. To model the blood pressure of the systemic circulation system during the ejection phase, the following two element Windkessel model is used (see e.g. [27])
  (2.4) $$C\frac{\text{d}P_{\text{art}}}{\text{d}t}+\frac{P_{\text{art}}}{R}=-\frac{\text{d}V_{\text{endo}}}{\text{d}t},$$
  where C defines the compliance, and R defines the impedance of the systemic circulation system. Here $P_{\text{art}}$ stands for arterial pressure.
• *Isovolumetric relaxation.* When the ventricular flow reverses, i.e. ${\overset{˙}{V}}_{\text{endo}}>0$, the isovolumetric relaxation phase begins. In this phase, the ventricular pressure decreases while keeping the ventricular volume constant at the end-systolic volume (ESV). The pressure is therefore obtained analogously to the isovolumetric contraction phase from (2.3).
• *Filling.* The isovolumetric relaxation phase ends when the pressure drops below a specified threshold. Blood flows from the atria to the ventricle while the ventricular volume returns to its initial value (end-diastolic volume). In this phase, the ventricular pressure is modelled with the following decay equation
  (2.5) $$\frac{\text{d}P_{\text{endo}}}{\text{d}t}=-\gamma \frac{\text{d}V_{\text{endo}}}{\text{d}t},$$
  where γ is the decay constant.

The left ventricular volume $V_{\text{endo}}$ is calculated throughout the cardiac cycle by using the divergence theorem and the assumption that the volume is constrained by a flat lid located on the basal plane [13], leading to

$$V_{\text{endo}}=\int_{\Omega_{\text{endo}}}\nabla_{x}\cdot \left[\left(x\cdot e_{1}\right)\;e_{1}\right]\;\text{d}\Omega =\int_{\partial \Omega_{\text{endo}}}\left(x\cdot e_{1}\right)\left(e_{1}\cdot n\right)\;\text{d}\partial \Omega =\int_{\partial \Omega_{0\;\text{endo}}}J\left(x\cdot e_{1}\right)\left(e_{1}\cdot F^{-\text{T}}n_{0}\right)\;\text{d}\partial \Omega_{0},$$

where $e_{1}=\left(1,0,0\right)$. Because of the orientation of the reference state of the ventricle and of its centreline, here we consider that $e_{1}\cdot n_{0}=0$ on the basal plane.

#### 2.2.2 Electrical propagation model

Simulation of propagation of electrical excitation was achieved through the monodomain equations [37] in the form:

(2.6a) $$\chi C_{\text{m}}\frac{\partial V}{\partial t}-\nabla_{X}\cdot \left(D_{0}\nabla_{X}V\right)+\chi I_{\text{ion}}\left(V,w,c\right)=\chi I_{\text{app}}\left(X,t\right),\;\text{in}\;\Omega_{0}\times \left(0,T\right],$$

(2.6b) $$\frac{\text{d}w}{\text{d}t}=m_{w}\left(V,w,c\right),\;\text{in}\;\Omega_{0}\times \left(0,T\right],$$

(2.6c) $$\frac{\text{d}c}{\text{d}t}=m_{c}\left(V,w,c\right),\;\text{in}\;\Omega_{0}\times \left(0,T\right],$$

where V is the transmembrane potential, w are the gating variables that regulate the transmembrane currents, c are the ionic concentrations inside of the cell, $m_{x}$ is the right-hand side of the system of ODEs corresponding to the generic state variable vector x, χ is the surface to volume ratio, $C_{\text{m}}$ is the membrane capacitance per unit area, $I_{\text{ion}}$ are the ionic currents, and $I_{\text{app}}$ is the applied current triggering the electrical depolarisation acting on specific anatomical locations. The orthotropic tensor of local conductivities in the reference configuration is defined as

(2.7) $$D_{0}=\sigma_{\text{f}}\;f_{0}⊗f_{0}+\sigma_{\text{s}}\;s_{0}⊗s_{0}+\sigma_{\text{n}}\;{\overset{¯}{n}}_{0}⊗{\overset{¯}{n}}_{0},$$

where $\sigma_{\text{f}}$, $\sigma_{\text{s}}$ and $\sigma_{\text{n}}$ are, respectively, the conductivities in the fibre, sheet and normal directions.

#### 2.2.3 Human-based ventricular cellular electromechanical model

The electrophysiological activity at the cellular level is encoded in (2.6b), (2.6c) and in the $I_{\text{ion}}$ term of (2.6a). Eqs. (2.6b), (2.6c) describe the membrane kinetics as well as the local transport of ions within cells. In this study, the modified version by [21] of the human ventricular cell electrophysiology model by O’Hara et al. [6] was used. It consists of a system of ODEs with 41 state variables, and it exhibits a highly stiff behaviour, mainly due to the activation of sodium ionic current, which causes a sudden spike in the transmembrane potential V. This is the human model that shows closer agreement with experimental recordings in a range of conditions and that has been adopted as the basis for the CiPA initiative for drug assessment, sponsored by the Food and Drug Association (FDA) for the *in-silico* [7].

Coupling of the electrophysiology to the contractile machinery of the cardiomyocytes is represented through the human-based model of excitation–contraction and active tension by [9]. The model is based on sets of ODEs describing the local dynamics of a vector of state variables q, which represents the contractile mechanisms in cardiomyocytes (see a detailed discussion about these models in [38]). The corresponding system of ODEs reads as (note the dependencies on stretch and stretch-rate)

(2.8) $$\frac{\text{d}q}{\text{d}t}=m_{q}\left(q,c,\lambda_{\text{f}},{\overset{˙}{\lambda }}_{\text{f}}\right).$$

where $\lambda_{\text{f}}=\sqrt{I_{4\text{f}}}=\sqrt{f_{0}\cdot Cf_{0}}$ is the stretch in the fibre direction. The Land et al. [9] model consists of six state variables $q=\left\{S,W,\text{CaTRPN},B,\zeta_{\text{s}},\zeta_{\text{w}}\right\}$: S and W are variables associated with the crossbridge binding, respectively being the post-powerstroke and pre-powerstroke states, CaTRPN represents the fraction of troponin C units with calcium bound to their regulatory binding site, B represents the fraction of blocked myosin binding sites on actin, and the state variables $\zeta_{\text{w}},\zeta_{\text{s}}$ dictate the pre-powerstroke and post-powerstroke distortion in a distortion-decay model.

The right-hand side terms defining $m_{q}$ in (2.8) assume the following form

$$m_{q}=\left(\begin{array}{c} k_{\text{ws}}W-k_{\text{su}}S-\gamma_{\text{su}}S\\ k_{\text{uw}}\left(1-B-S-W\right)-k_{\text{wu}}W-k_{\text{ws}}W-\gamma_{\text{wu}}W\\ k_{\text{TRPN}}\left[{\left(\frac{{\left[{\text{Ca}}^{2+}\right]}_{\text{int}}}{{\left[{\text{Ca}}^{2+}\right]}_{\text{T50}}}\right)}^{n_{\text{TRPN}}}\left(1-\text{CaTRPN}\right)-\text{CaTRPN}\right]\\ K_{\text{u}}{\text{CaTRPN}}^{-\frac{n_{\text{Tm}}}{2}}\left(1-B-S-W\right)-k_{\text{u}}{\text{CaTRPN}}^{\frac{n_{\text{Tm}}}{2}}B\\ A_{\text{s}}{\overset{˙}{\lambda }}_{\text{f}}-c_{\text{s}}\zeta_{\text{s}}\\ A_{\text{w}}{\overset{˙}{\lambda }}_{\text{f}}-c_{\text{w}}\zeta_{\text{w}} \end{array}\right),$$

where the state variable-dependent parameters are defined as

$$\gamma_{\text{su}}=\left\{\begin{array}{cc} \gamma_{\text{s}}\left(-\zeta_{\text{s}}-1\right)\;\; & \text{if}\;\zeta_{\text{s}}+1<0\\ \gamma_{\text{s}}\zeta_{\text{s}}\;\; & \text{if}\;\zeta_{\text{s}}+1>1\\ 0\;\; & \text{otherwise}, \end{array}\right)$$

$$\gamma_{\text{wu}}=\gamma_{\text{w}}\left|\zeta_{\text{w}}\right|,$$

$${\left[{\text{Ca}}^{2+}\right]}_{\text{T50}}={\left[{\text{Ca}}^{2+}\right]}_{\text{T50}}^{\text{ref}}+\beta_{1}\left[\text{min}\left(\lambda_{\text{f}},1.2\right)-1\right].$$

When the solution of the system of ODEs (2.8) is achieved, the expression for the active tension in the fibre direction $T_{\text{act}}$ can be retrieved as

(2.9) $$T_{\text{act}}\left(q,\lambda_{\text{f}}\right)=\overset{ˆ}{h}\left(\lambda_{\text{f}}\right)\frac{T_{\text{ref}}}{r_{\text{s}}}\left[\left(\zeta_{\text{s}}+1\right)S+\zeta_{w}W\right],$$

where

$$\overset{ˆ}{h}\left(\lambda_{\text{f}}\right)=\left\{\begin{array}{cc} 0\;\; & \text{if}\;\lambda_{\text{f}}<\frac{1.87\beta_{0}-1}{2\beta_{0}}\\ 1+\beta_{0}\left(2\lambda_{\text{f}}-1.87\right)\;\; & \text{if}\;\frac{1.87\beta_{0}-1}{2\beta_{0}}\leq \lambda_{\text{f}}<0.87\\ 1+\beta_{0}\left(\lambda_{\text{f}}-1\right)\;\; & \text{if}\;0.87\leq \lambda_{\text{f}}<1.2\\ 1+0.2\beta_{0}\;\; & \lambda_{\text{f}}\geq 1.2, \end{array}\right)$$

and the remaining parameters are constant.

Equations (2.6c), (2.8) are coupled bidirectionally, as specified by (2.8) and such that (2.6c) now becomes

$$\frac{\text{d}c}{\text{d}t}=m_{c}\left(V,w,c,q\right).$$

Particularly, the calcium bound to troponin needed by the cell electrophysiology model is obtained from the Land active tension model, which means that {c} depends on {q} as (a more complete derivation, following [6], and a specification of model constants are shown in Appendix B)

$$\frac{\text{d}{\left[{\text{Ca}}^{2+}\right]}_{\text{i}}}{\text{d}t}=\frac{1}{1+\frac{\left[\bar{\text{CMDN}}\right]K_{\text{CMDN}}}{{\left({\left[{\text{Ca}}^{2+}\right]}_{\text{i}}+K_{\text{CMDN}}\right)}^{2}}}\left[\;\;-\left({\text{I}}_{\text{pCa}}+{\text{I}}_{\text{Cab}}-2\;{\text{I}}_{\text{NaCa}}\right)\frac{{\text{A}}_{\text{cap}}}{2\;\text{F}\;{\text{v}}_{\text{myo}}}-\text{J}\frac{{\text{v}}_{\text{nsr}}}{{\text{v}}_{\text{myo}}}+{\text{J}}_{\text{Ca}}\frac{{\text{v}}_{\text{ss}}}{{\text{v}}_{\text{myo}}}-{\left[\text{TRPN}\right]}_{\text{max}}\frac{\text{dCaTRPN}}{\text{d}t}\right],$$

where ${\left[{\text{Ca}}^{2+}\right]}_{\text{i}}\in \left\{c\right\}$ and CaTRPN∈{q}. On the other hand, the calcium concentration needed by the active tension model is obtained from the cell electrophysiology model, i.e. {q} depends on {c} specifically through the evolution of the calcium bound to troponin [9], in the form

$$\frac{\text{dCaTRPN}}{\text{d}t}=k_{\text{TRPN}}\left[{\left(\frac{{\left[{\text{Ca}}^{2+}\right]}_{\text{i}}}{{\left[{\text{Ca}}^{2+}\right]}_{\text{T50}}}\right)}^{n_{\text{TRPN}}}\left(1-\text{CaTRPN}\right)-\text{CaTRPN}\right].$$

### 2.3 Description of the electromechanical coupling mechanisms

The electrophysiological and mechanical activities of the heart at the ventricular level are bidirectionally coupled through active stress (from electrical diffusion to solid deformation), the effect of deformation on diffusivity and stretch-activated ionic currents (the two latter are also known as mechano-electric feedback, i.e. from solid deformation to electrical diffusion).

#### 2.3.1 Active stress

As an active stress formulation is used, the stress term in (2.1a) is additively decomposed into passive and active terms, such that the balance of linear momentum reads

(2.10) $$\rho_{0}\left(X\right)\frac{\partial^{2}u\left(X\right)}{\partial t^{2}}-\nabla_{X}\cdot \left[F\left(S_{\text{pas}}+S_{\text{act}}\right)\right]=0,\;\text{in}\;\Omega_{0}\times \left(0,T\right].$$

Here the active Second Piola–Kirchhoff stress tensor is defined as

(2.11) $$S_{\text{act}}=JF^{-1}\sigma_{\text{act}}F^{-\text{T}}=JF^{-1}\left[\frac{1}{J}\left(\left(T_{\text{act}}+\sigma_{0}\right)\frac{Ff_{0}⊗Ff_{0}}{\lambda_{\text{f}}^{2}}+k_{\text{ort}\;1}T_{\text{act}}\frac{Fs_{0}⊗Fs_{0}}{\lambda_{\text{s}}^{2}}+k_{\text{ort}\;2}T_{\text{act}}\frac{F{\overset{¯}{n}}_{0}⊗F{\overset{¯}{n}}_{0}}{\lambda_{\text{n}}^{2}}\right)\right]F=\left(T_{\text{act}}+\sigma_{0}\right)\frac{f_{0}⊗f_{0}}{\lambda_{\text{f}}^{2}}+k_{\text{ort}\;1}T_{\text{act}}\frac{s_{0}⊗s_{0}}{\lambda_{\text{s}}^{2}}+k_{\text{ort}\;2}T_{\text{act}}\frac{{\overset{¯}{n}}_{0}⊗{\overset{¯}{n}}_{0}}{\lambda_{\text{n}}^{2}},$$

where $k_{\text{ort}\;1}$ and $k_{\text{ort}\;2}$ are activation parameters acting in the sheet and normal directions, respectively. These constants are taken as in [39], [40], [41], in which an orthotropic active stress tensor is assumed, considering that mechanical activation occurs differently in each local direction. The specific values of the weighting activation parameters can contribute to achieving physiological regimes of wall thickening and ejection fraction. It is also important to point out that in (2.11) the pre-stress given by (2.2) appears only in the fibre direction.

#### 2.3.2 Mechano-electric feedback

Mechano-electric feedback generally comprises of two coupling submechanisms in which the mechanics of cardiac tissue affect the electrophysiological activity. By adding stretch-activated ionic currents to the spatial formulation of (2.6a) and then pulling back to the reference configuration (see e.g. [42]), one obtains

(2.12) $$\chi C_{\text{m}}J\frac{\partial V}{\partial t}-\nabla_{X}\cdot \left(JF^{-1}DF^{-\text{T}}\nabla_{X}V\right)+\chi J\left[I_{\text{ion}}\left(V,w,c\right)+I_{\text{SAC}}\left(V,F\right)\right]=\chi JI_{\text{app}}\left(t\right),\;\text{in}\;\Omega_{0}\times \left(0,T\right].$$

The orthotropic tensor of local conductivities (2.7) is now defined as

(2.13) $$D=\sigma_{\text{f}}\frac{\left(Ff_{0}\right)⊗\left(Ff_{0}\right)}{\lambda_{\text{f}}^{2}}+\sigma_{\text{s}}\frac{\left(Fs_{0}\right)⊗\left(Fs_{0}\right)}{\lambda_{\text{s}}^{2}}+\sigma_{\text{n}}\frac{\left(F{\overset{¯}{n}}_{0}\right)⊗\left(F{\overset{¯}{n}}_{0}\right)}{\lambda_{\text{n}}^{2}},$$

where $\lambda_{\text{s}}=\sqrt{s_{0}\cdot Cs_{0}}$ and $\lambda_{\text{n}}=\sqrt{n_{0}\cdot Cn_{0}}$ are the stretches in the sheet and normal directions, respectively. The term $I_{\text{SAC}}$ in (2.12) denotes stretch-activated ionic currents defined as

(2.14) $$I_{\text{SAC}}\left(V,F\right)=g{\left(\lambda_{\text{f}}-1\right)}_{+}\left(V-E\right),$$

where E and g are, respectively, the reversal potential and the maximal conductance of the channels [43], [44]. Note that this term only becomes active when the tissue is stretched in the fibre direction, i.e. $\lambda_{\text{f}}>1$. In this study g is set to 0, regardless of the strain state.

It is worthy to note a few aspects of the chosen formulation (2.12)–(2.13). The pulled-back counterpart of the conductivity tensor defined on the spatial domain (2.13) is

(2.15) $$D_{0}=JF^{-1}DF^{\text{-T}}=J\left(\sigma_{\text{f}}\frac{f_{0}⊗f_{0}}{\lambda_{\text{f}}^{2}}+\sigma_{\text{s}}\frac{s_{0}⊗s_{0}}{\lambda_{\text{s}}^{2}}+\sigma_{\text{n}}\frac{n_{0}⊗n_{0}}{\lambda_{\text{n}}^{2}}\right),$$

which assumes that the conductivity coefficients have been measured in the spatial configuration. On the other hand, one could assume conductivity coefficients measured in the reference configuration, which would lead to the conductivity tensor in the reference configuration

(2.16) $$D_{0}=\sigma_{\text{f}}^{⋆}\;f_{0}⊗f_{0}+\sigma_{\text{s}}^{⋆}\;s_{0}⊗s_{0}+\sigma_{\text{n}}^{⋆}\;{\overset{¯}{n}}_{0}⊗{\overset{¯}{n}}_{0}.$$

As one can observe, the equivalences between the latter conductivity coefficients in (2.15), (2.16) are

$$\sigma_{\text{f}}^{⋆}=J\frac{\sigma_{\text{f}}}{\lambda_{\text{f}}^{2}},\;\sigma_{\text{s}}^{⋆}=J\frac{\sigma_{\text{s}}}{\lambda_{\text{s}}^{2}},\;\text{and}\;\sigma_{\text{n}}^{⋆}=J\frac{\sigma_{\text{n}}}{\lambda_{\text{n}}^{2}},$$

which means that if the conductivity coefficients are fixed in one configuration, they vary in the other configuration according to the corresponding strain distribution. In particular, the formulation chosen for this study (2.12)–(2.13) assumes conductivity coefficients measured in the spatial configuration; this means that, for instance, under an isovolumetric extension in the fibre direction

$$F=\lambda^{-\frac{1}{2}}I+\left(\lambda -\lambda^{-\frac{1}{2}}\right)f_{0}⊗f_{0},$$

the conductivity coefficient in the reference configuration $\sigma_{\text{f}}^{⋆}$ would be reduced by a factor $\lambda^{2}$. A more exhaustive description of the effect of deformation on diffusivity, its different formulations and implications can be seen in [13].

### 2.4 Weak formulation

The first step towards obtaining a numerical solution of the proposed nonlinear coupled problem is to define an adequate weak formulation of Eqs. (2.10), (2.1b), (2.1c), (2.12), (2.6b), (2.6c), (2.8), posed in the reference configuration. Assuming sufficient regularity for initial conditions and source terms, for a fixed time, one proceeds to contract the governing equations by sufficiently regular test functions, to integrate by parts the divergence terms, and to use appropriately the boundary conditions to obtain the following variational problem:

For t>0, find $\left(u\left(t\right),V\left(t\right),q\left(t\right),w\left(t\right),c\left(t\right)\right)\in H\times V\times Q^{n_{q}}\times Q^{n_{w}}\times Q^{n_{c}}$ (where the power $n_{i}$ denotes the dimension of the vector of each species i∈{q,w,c}), such that

(2.17a) $$\int_{\Omega_{0}}\rho_{0}\frac{\partial^{2}u\left(t\right)}{\partial t^{2}}\cdot v\;\text{d}\Omega_{0}+\int_{\Omega_{0}}FS:\nabla_{X}v\;\text{d}\Omega_{0}-\int_{\partial \Omega_{0\;\text{epi}}}\left[k_{\text{epi}}\;u\left(t\right)\cdot n_{0}\right]n_{0}\cdot v\;\text{d}\partial \Omega_{0}\;=\int_{\partial \Omega_{0\;\text{endo}}}JP_{\text{endo}}F^{-\text{T}}n_{0}\cdot v\;\text{d}\partial \Omega_{0},\;\forall v\in H,$$

(2.17b) $$\chi C_{\text{m}}\int_{\Omega_{0}}J\frac{\partial V\left(t\right)}{\partial t}\varphi \;\text{d}\Omega_{0}-\int_{\Omega_{0}}JF^{-1}DF^{-\text{T}}\nabla_{X}V\left(t\right)\cdot \nabla_{X}\varphi \;\text{d}\Omega_{0}\;+\chi \int_{\Omega_{0}}JI_{\text{ion}}\left(V\left(t\right),w\left(t\right),c\left(t\right),q\left(t\right)\right)\;\varphi \;\text{d}\Omega_{0}+\chi \int_{\Omega_{0}}\;\;JI_{\text{SAC}}\left(V\left(t\right),F\right)\varphi \;\text{d}\Omega_{0}\;=\chi \int_{\Omega_{0}}\;\;JI_{\text{app}}\left(X,t\right)\varphi \;\text{d}\Omega_{0},\;\forall \varphi \in V,$$

(2.17c) $$\int_{\Omega_{0}}\left[\frac{\text{d}q}{\text{d}t}-m_{q}\left(q,c,\lambda_{\text{f}},{\overset{˙}{\lambda }}_{\text{f}}\right)\right]\cdot r\;\text{d}\Omega_{0}=0,\;\forall r\in Q^{n_{q}},$$

(2.17d) $$\int_{\Omega_{0}}\left[\frac{\text{d}w}{\text{d}t}-m_{w}\left(V,w,c\right)\right]\cdot s\;\text{d}\Omega_{0}=0,\;\forall s\in Q^{n_{w}},$$

(2.17e) $$\int_{\Omega_{0}}\left[\frac{\text{d}c}{\text{d}t}-m_{c}\left(V,w,c,q\right)\right]\cdot d\;\text{d}\Omega_{0}=0,\;\forall d\in Q^{n_{c}},$$

where the functional spaces of the test functions (v,φ,r,s,d) in (2.17a)–(2.17b), (2.17c)–(2.17e) are respectively specified as

$$H=H^{1}\left(\Omega_{0},R^{3}\right),\;V=H^{1}\left(\Omega_{0},R\right),\;Q=L^{2}\left(\Omega_{0},R\right),$$

and note that these coincide with the trial spaces in (2.17a), (2.17b) since no part of the boundary assumes essential boundary conditions.

The problem under consideration is highly nonlinear and multiscale, and it features intricate interdependencies between submodels. Obtaining approximate solutions based on fully monolithic approaches is therefore not feasible as the number of state variables of the physiologically-relevant cell electrophysiology models can be large (frequently these models also feature Heaviside functions, which compromise the well-definiteness of Jacobian operators). Instead, a semi-implicit partitioned approach is proposed, and is made precise in what follows. The use of segregated approaches is additionally advantageous as the propagation of electrical depolarisation consists of a moving wave with a steep wavefront (with a width of fractions of millimetre, and with a duration of the upstroke of the action potential of ∼2 ms), while the tissue deformation does not exhibit such a particularly local behaviour [45], [46]. This would allow, for instance, the use of different meshes: a finer mesh for electrical propagation and a coarser mesh for solid mechanics, although this has not been done in this study.

The detailed description of the numerical implementation of the model, including the finite element discretisation, the time discretisation, operator splitting and subsequent linearisation can be found in Appendix D, Appendix E.

### 2.5 Software and simulation platform

The software used in this study is Alya, developed at the Barcelona Supercomputing Center (BSC) [14], [17], [18], [20], [47]. Alya is a multi-physics code that works in parallel, specially designed to efficiently simulate strongly coupled problems in large-scale HPC platforms. Electrical diffusion and mechanical deformation are both solved in the same code instance, in a staggered manner. Parallelisation is based on a hybrid message passing interface (MPI)/OpenMP paradigm, in which the mesh is partitioned at a pre-processing stage by using METIS [48]. With respect to its scalability, an example can be found in [19], where the largest simulation so far of a cardiac electromechanical model of a biventricular geometry is performed, with a mesh consisting of 3.4 billion tetrahedra, reaching an almost linear scalability up to 100,000 cores in NCSA’s Blue Waters. Verification tests have been conducted as part of previous studies [20], [47].

The 3D ventricular simulations were run on the supercomputer MareNostrum IV (BSC), by using 2400 MPI processes (equivalent to 2400 computing cores), and would take from 2 to 40 hours (including the initiation phase), depending on the considered time step. The assigned computational time was enough to ensure that both isovolumetric contraction and ejection phases were completed for a single heartbeat. This enables calculation of the considered clinically-relevant mechanical biomarkers.

**Initiation of the simulations.** The human-based coupled electromechanical cellular model was iterated in a single instance before starting the FE simulation itself. The pacing was chosen as 70 bpm during 1000 beats, the latter being, according to previous experience by the authors, enough to bring a cellular model of similar characteristics to a “periodic steady-state” [21]. Stretch and stretch rate were respectively set to 1 and 0 during the initiation of the cellular model. The results of the state variables of the cell electrophysiology model {w,c} were assigned to each node of the FE mesh as their initial values $\left\{w_{0},c_{0}\right\}$; likewise, the results of the state variables of the active tension model q were assigned to each integration point of the FE mesh (as their initial values $q_{0}$). The activation sequence of the reaction–diffusion starts with the activation of the endocardium and then progresses by diffusion to the rest of the myocardium. A current stimulus was applied for 2 ms, which enabled activation of the ventricle.

**Model parameters.** Myocardial tissue was modelled as an orthotropic material for both mechanical deformation and electrical diffusion. The corresponding diffusivity values were 0.001 cm${}^{2}∕\mathrm{ms}$, 0.0005 cm${}^{2}∕\mathrm{ms}$ and 0.00025 cm${}^{2}∕\mathrm{ms}$ for, respectively, the fibre, sheet and normal directions [49]. Fibre directions are parallel to the basal plane in the middle of the cardiac wall, and vary linearly up to forming an angle ±ϕ with the basal plane at the endocardium/epicardium [50], and tangent to the circumferential direction. Sheet directions are parallel to the vectors normal to the endocardial/epicardial surface, and do not vary throughout the cardiac wall. Other parameter values will be detailed in Section 2.6.

### 2.6 Sensitivity analysis

#### 2.6.1 Choice of sensitivity parameters

The parameters chosen for the sensitivity analysis were the following: stiffness of the epicardium Robin boundary condition $k_{\text{epi}}$, end-diastolic pressure $P_{0}$, pressure at which the ejection phase is triggered $P_{\text{ej}}$, compliance of the 2-elements Windkessel model C, resistance of the 2-elements Windkessel model R, orthotropic activation parameter in the normal direction $k_{\text{ort}\;2}$, active stress scaling parameter of the active tension model $T_{\text{ref}}$, the angle of fibre distribution ϕ, bulk modulus K, and the linear parameter (i.e. that with stress magnitude) of the passive strain energy density function in the sheet direction $a_{\text{s}}$. These parameters were chosen amongst the whole set of mechanical parameters due to a combination of the following factors: (1) uncertainty of their values; (2) feasibility of the study; (3) their effect on the mechanical biomarkers in cellular and tissue sensitivity studies. With respect to passive properties, simulations were carried out to investigate which of the mechanical parameters of the strain energy density function had a substantial impact on the mechanical biomarkers. For this, the so-called linear parameters (those with stress dimension) in the Holzapfel–Ogden strain energy density function [34] were considered. Variations of 50%–200% in single parameters with respect to the baseline values were tested, and only $a_{\text{s}}$ was found to result in variations larger than 5% in EF. Furthermore, in the cellular active contraction model, $T_{\text{ref}}$ was identified as the most critical parameter for active tension, and therefore was considered in the sensitivity analysis.

#### 2.6.2 General approach

The sensitivity analysis was divided into two parts: a one-at-a-time approach and a partial rank correlation coefficient (PRCC) sampling-based global approach. One-at-a-time approaches involve variation of a single parameter while keeping the rest fixed. Sampling-based approaches involve the generation and exploration of a mapping from the model parameters, sampled in this study by Latin Hypercube Sampling (LHS) [51], to the desired mechanical biomarkers (known in the uncertainty quantification and sensitivity analysis literature as “quantities of interest”) [52]. Then, the importance of the individual parameters with respect to the uncertainty in the mechanical biomarkers is assessed by the analysis of the PRCC, as proposed in [53], [54]. Uniform probability density distributions were assumed for the considered input parameters, as parameter distributions are unknown [55]. PRCC-based sensitivity analysis studies are not new in the context of cardiac electrophysiology [56], [57], [58].

Correlation provides a measure of the relationship between model input parameters and output biomarkers. A correlation coefficient (CC) $c\left(x_{j},y\right)$ between an input parameter $x_{j}$ and a biomarker y is

(2.18) $$c\left(x_{j},y\right)=\frac{\overset{n_{\text{sam}}}{\underset{i=1}{\sum }}\left(x_{ij}-{\overset{¯}{x}}_{j}\right)\left(y_{i}-\overset{¯}{y}\right)}{\sqrt{\overset{n_{\text{sam}}}{\underset{i=1}{\sum }}{\left(x_{ij}-{\overset{¯}{x}}_{j}\right)}^{2}}\sqrt{\overset{n_{\text{sam}}}{\underset{i=1}{\sum }}{\left(y_{i}-\overset{¯}{y}\right)}^{2}}},$$

where

$${\overset{¯}{x}}_{j}=\frac{1}{n_{\text{sam}}}\overset{n_{\text{sam}}}{\underset{i=1}{\sum }}x_{ij};\;\overset{¯}{y}=\frac{1}{n_{\text{sam}}}\overset{n_{\text{sam}}}{\underset{i=1}{\sum }}y_{i}.$$

The CC $c\left(x_{j},y\right)$ varies from −1 to +1, where a positive value would indicate that $x_{j}$ and y tend to increase, or decrease, together and a negative value would indicate the opposite; a value of 0 indicates the presence of no linear relationship. The partial correlation coefficient (PCC) between $x_{j}$ and y can be defined as follows. First, the regression models

xˆj=c0+∑p=1p≠jnparcpxpandyˆ=b0+∑p=1p≠jnparbpxp

are constructed. The results of these linear regressions are used to defined the new variables $x_{j}-{\overset{ˆ}{x}}_{j}$ and $y-\overset{ˆ}{y}$. Partial correlation characterises the linear relationships between an input parameter $x_{j}$ and a biomarker y after a correction for the linear effects on y of the remaining input parameters is made. The PCC between $x_{j}$ and y are defined as the CC (2.18) between $x_{j}-{\overset{ˆ}{x}}_{j}$ and $y-\overset{ˆ}{y}$, i.e. $c\left(x_{j}-{\overset{ˆ}{x}}_{j},y-\overset{ˆ}{y}\right)$. Similarly to PCC, partial rank correlation performs correlation on rank-transformed data, which means that $x_{j}$ and y are replaced by their corresponding ranks (e.g. the smallest value is assigned the rank of 1, the following largest value is assigned the rank of 2, up to the largest value, which is assigned the rank of $n_{\text{sam}}$). PRCC is a robust sensitivity coefficient for nonlinear but monotonic relationships between $x_{j}$ and y, as long as little to no correlation exists between the input parameters [53], [59], [60].

A one-at-a-time approach was considered for the following two reasons, (1) as a preliminary exploration of the parameter space, with the purpose of approximately assessing the potential monotonicity and nonlinearity of the mechanical biomarkers with respect to each individual model parameter; (2) to probe suitable ranges of model parameters for the subsequent PRCC-based global sensitivity analysis. Thus simulations varying one parameter at a time informed the global approach for the sensitivity analysis using LHS. The one-at-a-time approach considered ten values for each of the parameters, while keeping the others constant. Therefore, a total number of samples $n_{\text{sam}}$ equal to 100, ten samples per parameter, was considered. These values for the parameters were obtained as

$$x_{i}=A_{i}+\left(j-1\right)\frac{B_{i}-A_{i}}{n_{\text{val}}-1},\;i=1,2,\ldots ,n_{\text{par}},\;j=1,2,\ldots ,n_{\text{val}}.$$

where $A_{i}$ and $B_{i}$ are, respectively, the lower and upper bounds of the parameter ranges. A summary of the parameters included in the sensitivity analysis and the ranges of considered values are shown in Table 2.1; the baseline values of these parameters are shown in Table 2.2. These ranges of values generally extended from 50% of the baseline value to 200%–1000% of the baseline value, with the exception of $k_{\text{epi}}$, whose values were completely unknown; $k_{\text{ort}\;2}$, whose maximum value was obtained from [40], [41] and not increased further due to the stability of the numerical treatment (a higher value of this parameter could compromise the positive-definiteness of the tangent operator, hampering the convergence of the Newton–Raphson scheme); and the fibre angle ϕ, whose values were directly obtained from the estimations made in [50].

The sampling of the parameter space for the global sensitivity analysis considered LHS, with the limits defined by the ranges of values in Table 2.1. A number of samples equal to 10 times the number of parameters were also considered, leading to 100 samples (200 in total). This number of LHS samples was deemed suitable as there are studies in the literature that used a number of LHS samples of the same order for a much higher number of independent parameters [61], [62].

**Table 2.1:** Parameters included in the sensitivity analysis, their ranges of values, units, (sub)model where they belong, the domain to which the corresponding (sub)model is applied to, and reference(s) from where the ranges of values are inferred.

Parameter	Range of values	Units	(Sub)Model	Applied to	Ref.
$k_{\mathrm{epi}}$	0–1,000,000	Ba cm^−1^	Robin	$\partial \Omega_{0\;\mathrm{epi}}$	N/A
$P_{0}$	7.5–30	mmHg	Windkessel	$\partial \Omega_{0\;\mathrm{endo}}$	[24], [27]
$P_{\mathrm{ej}}$	33.75–135	mmHg	Windkessel	$\partial \Omega_{0\;\mathrm{endo}}$	[24]
C	0.1–0.4	ml mmHg^−1^	Windkessel	$\partial \Omega_{0\;\mathrm{endo}}$	[27]
R	75–750	mmHg ms ml^−1^	Windkessel	$\partial \Omega_{0\;\mathrm{endo}}$	[27]
$k_{\mathrm{ort}\;2}$	0.0–0.6	N/A	Orthotropic activation	$\Omega_{0}$	[40]
$T_{\mathrm{ref}}$	60–1200	kPa	Active stress	$\Omega_{0}$	[9]
ϕ	45–90	deg	Fibre distribution	$\Omega_{0}$	[50]
K	500–5000	kPa	Passive stress	$\Omega_{0}$	[27]
$a_{s}$	512.8–7692	Pa	Passive stress	$\Omega_{0}$	[27]

**Table 2.2:** Values of the baseline parameters.

Parameter	$k_{\mathrm{epi}}$ (Ba cm^−1^)	$P_{0}$ (mmHg)	$P_{\mathrm{ej}}$ (mmHg)	C (ml⋅mmHg^−1^)	K (kPa)
Value	200,000	20	67.5	0.2	3333

Parameter	R (mmHg⋅ms⋅ml^−1^)	$k_{\mathrm{ort}\;2}$	$T_{\mathrm{ref}}$ (kPa)	ϕ (deg)	$a_{s}$ (Pa)
Value	750	0	120	60	2564

#### 2.6.3 Choice of mechanical biomarkers

The present electromechanical simulations aim to reproduce clinically-relevant ventricular activity (and to then be further used for simulations under diseased conditions or drug action). A suitable set of outputs or mechanical biomarkers was chosen. These included ejection fraction (EF), which represents the amount of blood that is pumped during a heartbeat

$$\text{EF}=100\frac{\text{EDV}-\text{ESV}}{\text{EDV}},$$

end-systolic pressure (ESP), which is the maximum ventricular pressure achieved in the left ventricle during a heartbeat; longitudinal fractional shortening (LFS) [63], which is a fractional version of the relative displacement between the endocardial apex and the base. LFS is evaluated as

$$\text{LFS}=100\frac{{\text{L}}_{0}-\text{L}}{{\text{L}}_{0}},$$

where L and ${\text{L}}_{0}$ are, respectively, the endocardial apico-basal distance at the end of the ejection phase and the initial (or end-diastolic) endocardial apico-basal distance (i.e. the “length” of the cavity of the truncated ellipsoid); and wall thickening (WT) [64], which is the fractional version of the relative displacement between points in the endocardium and epicardium that are at the same position relative to the apico-basal axis

$$\text{WT}=100\frac{\text{T}-{\text{T}}_{0}}{{\text{T}}_{0}},$$

where T and ${\text{T}}_{0}$ are, respectively, the cardiac wall thickness evaluated at the end of the ejection phase and the initial cardiac wall thickness. T and ${\text{T}}_{0}$ are evaluated in this study via the displacements of two points which are at the same angle relative to the apico-basal axis and at the same height relative to the endocardial apex, at the beginning of the simulation, one of them is on the endocardium and the other on the epicardium; the thickness values were obtained as the difference of the distance of these points to the apico-basal axis.

The physiological ranges of these mechanical biomarkers for healthy subjects are shown in Table 2.3.

**Table 2.3:** Mechanical biomarker values for healthy subjects. Values are shown as either full ranges, or 95% confidence interval; if the reference showed mean ± SD, these were converted to 95% confidence intervals through mean±1.96SD. Experimental data distributions are assumed to be normal to allow for such extrapolation.

Mechanical biomarker	Values	Units	Ref.
EF	48–69	%	[33]
ESP	100–174	mmHg	[65]
LFS	13–21	%	[66]
WT	18–100	%	[67]

## 3 Results

### 3.1 Effect of varying one-at-a-time model parameters on mechanical biomarkers

**Fig. 3.1:**
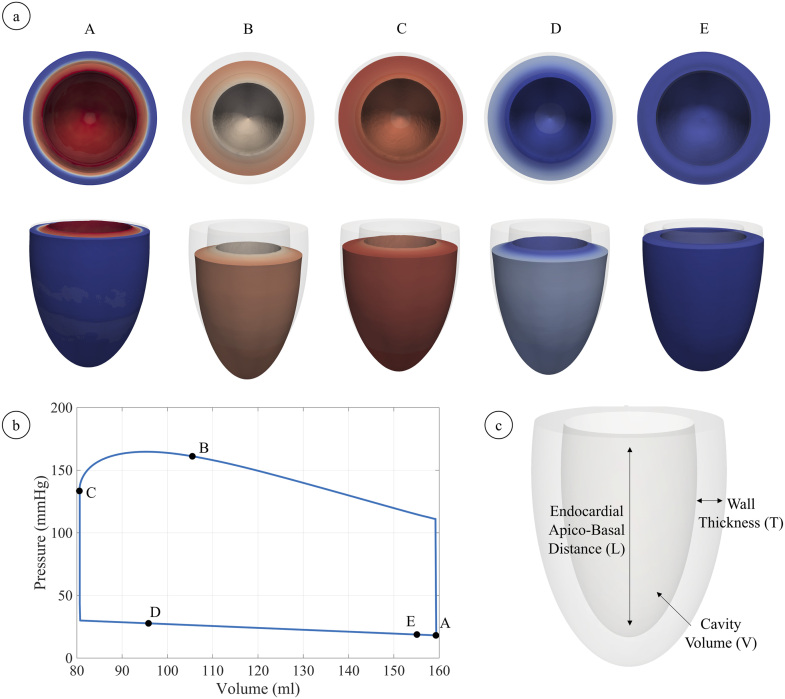
**(a)** Sequence of snapshots of contraction throughout the whole heartbeat, the colour code indicates transmembrane potential (red, fully activated and depolarised, and blue, polarised); **(b)** pressure–volume loop throughout the whole heartbeat, A–E correspond to the snapshots in **(a)**; **(c)** a sketch depicting wall thickness and apico-basal distance (which are the absolute counterparts of wall thickening (WT) and longitudinal fractional shortening (LFS)). (For interpretation of the references to colour in this figure legend, the reader is referred to the web version of this article.)

A typical pressure–volume curve showing a complete heartbeat is shown in Fig. 3.1b; different snapshots of ventricular morphology during such heartbeat are shown (Fig. 3.1a). A visual representation of WT and LFS is also shown to aid the reader in their visual representation (Fig. 3.1c). As expected, the volume during isovolumetric contraction remains constant with the corresponding pressure build up. A reduction in volume and a peak in pressure during the ejection phase follow. When the ventricle starts relaxing and the volume flow reverses, isovolumetric relaxation begins, which implies constant volume and a further reduction in pressure while the myocardium continues repolarising. The final phase, filling phase leads to the slow recovery of the initial volume while intraventricular pressure decreases.

**Fig. 3.2:**
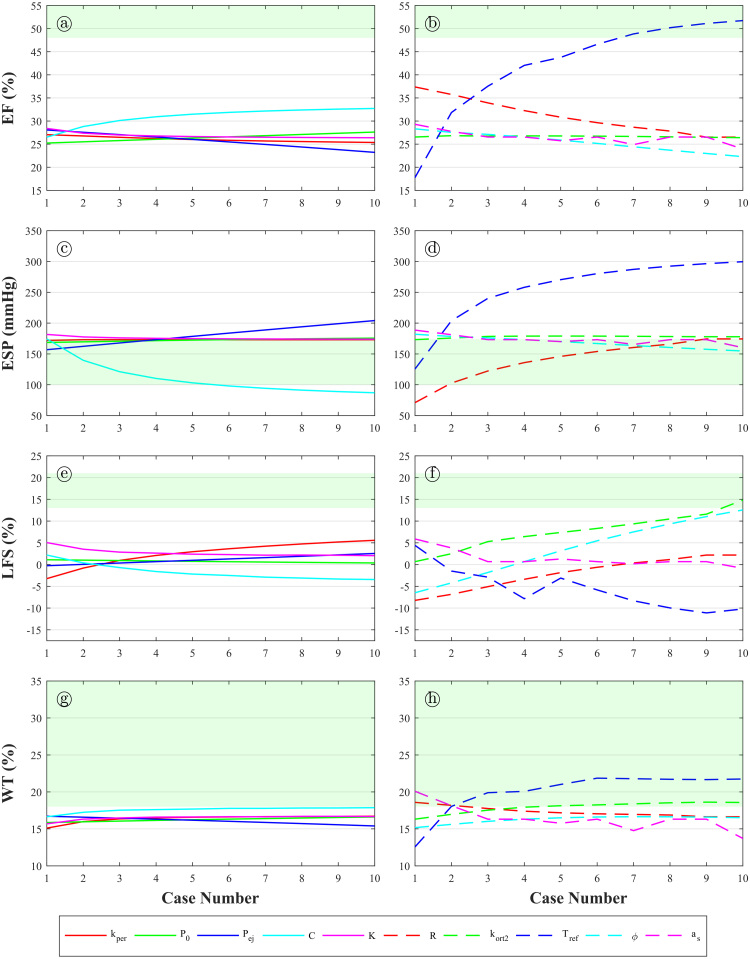
Mechanical biomarkers versus one-at-a-time variations of the considered parameters. **(a)** Ejection fraction versus $\left\{k_{\text{epi}},P_{0},P_{\text{ej}},C,K\right\}$. **(b)** Ejection fraction versus $\left\{R,k_{\text{ort}\;2},T_{\text{ref}},\phi ,a_{\text{s}}\right\}$. **(c)** End-systolic pressure versus $\left\{k_{\text{epi}},P_{0},P_{\text{ej}},C,K\right\}$. **(d)** End-systolic pressure versus $\left\{R,k_{\text{ort}\;2},T_{\text{ref}},\phi ,a_{\text{s}}\right\}$. **(e)** Longitudinal fractional shortening versus $\left\{k_{\text{epi}},P_{0},P_{\text{ej}},C,K\right\}$. **(f)** Longitudinal fractional shortening versus $\left\{R,k_{\text{ort}\;2},T_{\text{ref}},\phi ,a_{\text{s}}\right\}$. **(g)** Wall-thickening versus $\left\{k_{\text{epi}},P_{0},P_{\text{ej}},C,K\right\}$. **(h)** Wall-thickening versus $\left\{R,k_{\text{ort}\;2},T_{\text{ref}},\phi ,a_{\text{s}}\right\}$. Areas shaded in green represent the healthy ranges of the considered mechanical biomarkers. (For interpretation of the references to colour in this figure legend, the reader is referred to the web version of this article.)

 The values of the mechanical biomarkers for the one-at-a-time variations ($1,2,\ldots ,n_{\text{val}}$) of the model parameters ($1,2,\ldots ,n_{\text{par}}$) are shown in Fig. 3.2, with the clinically-reported healthy ranges of the mechanical biomarkers shown in green. It can be observed that the relationships between parameters and biomarkers are monotonic (and nonlinear) in almost all of the cases. It can be seen that the parameter which affects the mechanical biomarkers more strongly is $T_{\text{ref}}$ (Fig. 3.2b, c, f, h), with a particularly strong effect on EF (Fig. 3.2b). By just varying this parameter to high values, healthy values of EF (>48%) are attained. Other relationships shown to be important are EF−C (Fig. 3.2a), EF−R (Fig. 3.2b), $\text{ESP}-P_{\text{ej}}$ (Fig. 3.2c), ESP−C (Fig. 3.2c), ESP−R (Fig. 3.2d), $\text{LFS}-k_{\text{epi}}$ (Fig. 3.2e), $\text{LFS}-k_{\text{ort}\;2}$ (Fig. 3.2f), and LFS−ϕ (Fig. 3.2f).

**Fig. 3.3:**
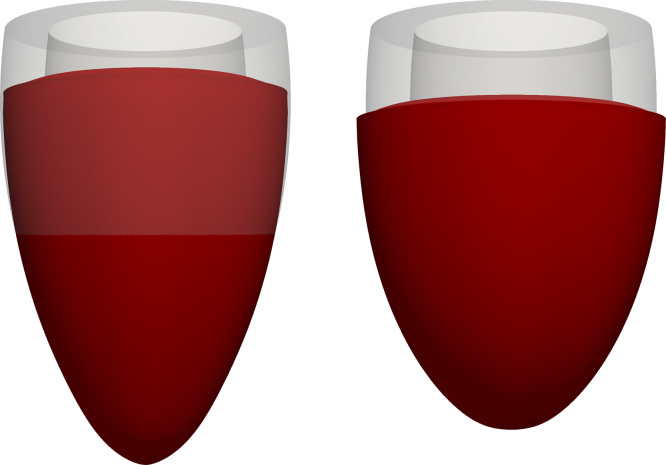
**(Left)** Mechanical deformation of the left ventricular geometry from diastole (transparent grey) to systole (red) for two cases with different mechanical parameter values. Left panel corresponds to low pericardial stiffness ($k_{\text{epi}}$) or low fibre angle (ϕ), whereas right panel results from high $k_{\text{epi}}$ or high ϕ. (For interpretation of the references to colour in this figure legend, the reader is referred to the web version of this article.)

Results show that increasing $P_{\text{ej}}$ increases the haemodynamic load of the left ventricle and delays the transition between isovolumetric contraction and ejection phases, directly affecting the maximum pressure obtained during a cardiac cycle (i.e. directly increasing ESP, Fig. 3.2c). Increasing C and R has opposite effects. The haemodynamic load of the ventricle is reduced, thus making the ventricle more compliant, as C is increased or R is decreased; this leads to increased EF (Fig. 3.2a–b) and decreased ESP (Fig. 3.2c–d). By just modifying one of these two parameters, healthy values of ESP can be attained (Fig. 3.2c–d). Increasing $k_{\text{epi}}$ leads to a higher stiffness of the pericardium, which is the membrane surrounding the heart; LFS is increased (Fig. 3.2e) because of the restricted motion of the apex and the resulting “sliding”-like contractile motion (i.e. the epicardial surface slides over its initial configuration, Fig. 3.3). The parameter ϕ (the angle between the basal plane and the fibre direction in the epicardial/endocardial surface) has a similar effect on LFS. Increasing ϕ leads to the active tension distribution in the fibre direction to more closely follow the apico-basal direction in the endocardium and epicardium, causing an increase in apico-basal motion (and thus an increased LFS, Fig. 3.2f). Healthy values of LFS are not achieved by one-at-a-time variations of the considered input parameters (Fig. 3.2e–h). The parameter $k_{\text{ort}\;2}$ is the fraction of active tension in the fibre direction which is apportioned to the ${\overset{¯}{n}}_{0}$ direction. The ${\overset{¯}{n}}_{0}$ direction is normal to the fibre-sheet plane and the sheet direction is parallel to the epicardial/endocardial surface normal. Higher $k_{\text{ort}\;2}$ leads to higher tension in the apico-basal direction throughout the whole ventricle, increasing the apico-basal motion in a similar fashion to when increasing ϕ (and therefore, increasing LFS, Fig. 3.2f).

### 3.2 Global sensitivity analysis results

**Fig. 3.4:**
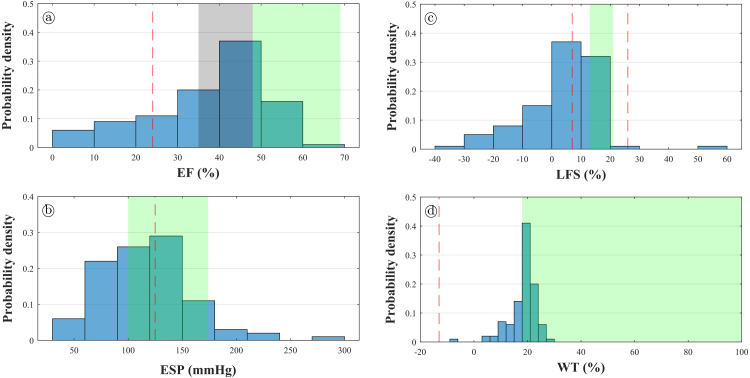
Histograms of simulated mechanical biomarkers compared to clinical values. The green shaded area indicates clinical ranges for healthy patients (Table 2.3). **(a)** Ejection fraction. The grey shaded area corresponds to low ejection fraction (<35% is the threshold for implantable cardioverter-defibrillator indicated by clinical guidelines [68]). The dashed red line indicates the clinical mean value for heart failure patients. [72]. **(b)** End-systolic pressure. The dashed red line indicates the clinical mean value for dilated cardiomyopathy patients [65]. **(c)** Longitudinal fractional shortening. The dashed red lines indicate the clinical mean values for patients with coronary artery disease [73], left, and high blood pressure with supranormal ejection fraction [74], right. **(d)** Wall thickening. The dashed red line corresponds to the clinical mean value for patients with ischaemic heart disease with dyskinetic segments [75]. (For interpretation of the references to colour in this figure legend, the reader is referred to the web version of this article.)

The combined effect of varying the ten model parameters on the mechanical biomarkers was evaluated via a global PRCC- and LHS-based sampling approach. The histograms showing the probabilistic distribution of the considered mechanical biomarkers are shown in Fig. 3.4. The shaded green area corresponds to the healthy range of the corresponding biomarker, shown in Table 2.3. The grey shaded area corresponds to the low ejection fraction zone (<35% is the threshold for implantable cardioverter-defibrillator under certain clinical guidelines [68]). The dashed red lines are mean values for exemplar diseases. As it can be noted, the obtained values for the mechanical biomarkers show a skewed distribution. The peak of the distribution of EF lies on the lower range of healthy values; the peak of the distribution of ESP lies in the middle of the range of healthy values; and the peak of the distribution of LFS lies between the mean of the diseased values and the lower threshold for healthy values. Therefore, the developed numerical model can reproduce a wide variety of outcomes, including those typically considered “diseased”, and those within healthy conditions. The obtained values of WT are 18.34±5.29%. Although this range of values does intersect the healthy range shown in Table 2.3, these values lie on the lower range of healthy values. The healthy ranges of WT in literature have a considerable spread [64], [69], [70]; even within the same study the healthy range can vary enormously due to the considered method of evaluation [70], due to inter-individual variability [67], or even due to intra-individual variability [71]. Such sparsity of WT ranges in the literature does not allow for a straightforward comparison with the results obtained in the present study.

**Fig. 3.5:**
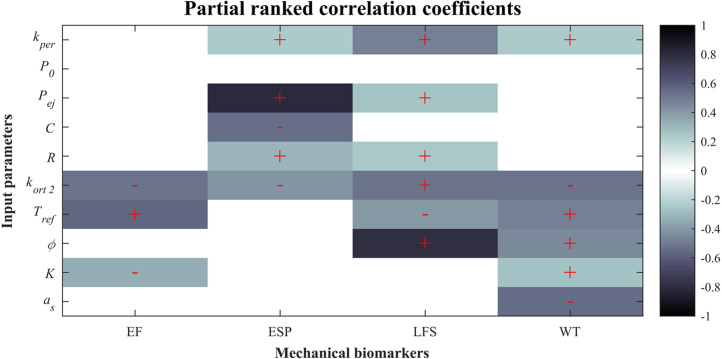
Correlation plot showing the partial rank correlation coefficients between each of the varied parameters and each of the simulated mechanical biomarkers. White grids have almost zero correlation coefficients and thus an associated p-value lower than the threshold for statistical significance (p<0.05). “Plus” or “minus” symbols only appear in the statistically significant values of the correlation coefficients and indicate a positive or a negative correlation, respectively.

Fig. 3.5 shows the PRCC values which are statistically significant (p≤0.05); “plus” or “minus” symbols are included in the significant PRCC, indicating a positive or a negative correlation, respectively. Results are consistent with the sensitivity analysis varying just one parameter at a time (Fig. 3.2). EF is positively correlated with $T_{\text{ref}}$ (moderate correlation) and negatively correlated with $k_{\text{ort}\;2}$ (moderate correlation); ESP is positively correlated with $P_{\text{ej}}$ (strong correlation) and negatively correlated with C (moderate correlation); LFS is positively correlated with $k_{\text{epi}}$ (weak correlation), $k_{\text{ort}\;2}$ (weak correlation), and ϕ (strong correlation); WT is negatively correlated with $k_{\text{ort}\;2}$ and $a_{\text{s}}$ (both with moderate correlation); all the other statistically significant correlation coefficients are relatively weak in comparison with the mentioned ones, i.e. closer to zero. Fig. 3.5 also suggests that EF, ESP and LFS could be dominated by mainly two parameters each.

**Fig. 3.6:**
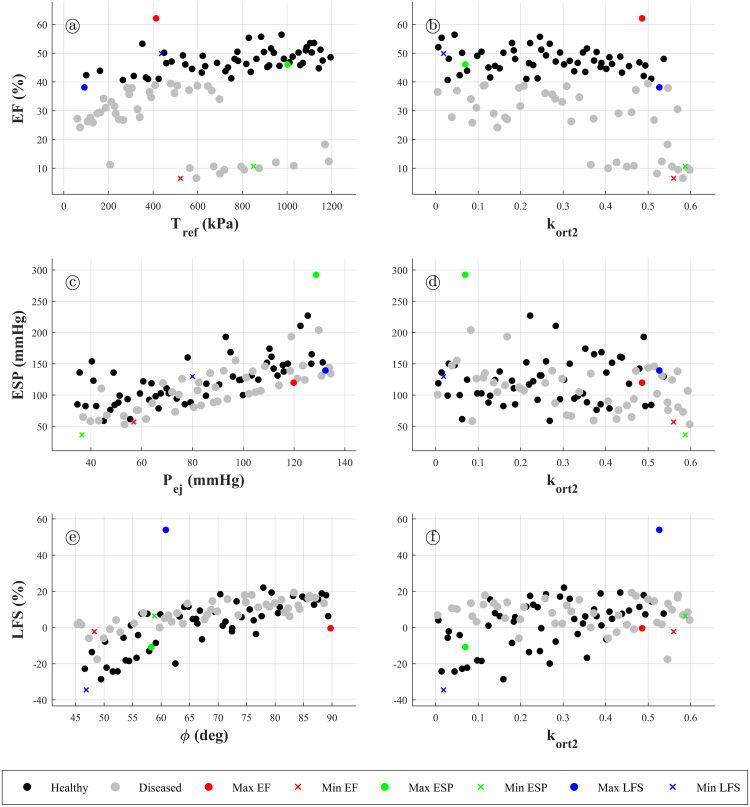
Scatter-plots of the mechanical biomarkers with respect to their most influential model parameters. **(a)** Ejection fraction versus $T_{\text{ref}}$. **(b)** Ejection fraction versus $k_{\text{ort}\;2}$. **(c)** End-systolic pressure versus $P_{\text{ej}}$. **(d)** End-systolic pressure versus $k_{\text{ort}\;2}$. **(e)** Longitudinal fractional shortening versus ϕ. **(f)** Longitudinal fractional shortening versus $k_{\text{ort}\;2}$. Black circles denote “healthy” cases (ejection fraction higher than 40%), whereas grey circles denotes “diseased” cases (ejection fraction lower than 40%). Coloured circles denote cases that maximise a mechanical biomarker (red for ejection fraction, green for end-systolic pressure and blue for longitudinal fractional shortening). On the other hand, coloured crosses denote cases that minimise a mechanical biomarker. (For interpretation of the references to colour in this figure legend, the reader is referred to the web version of this article.)

Fig. 3.6 shows the values of EF, ESP and LFS with respect to their most influential parameters according to the PRCC values in Fig. 3.5. EF is the most important mechanical biomarker, as it is usually employed to classify cardiac function into healthy or non-healthy. In this figure, a distinction between “healthy” cases (EF ≥ 40%) and “diseased” cases (EF < 40%) is made for graphical purposes, according to clinical guidelines [76]. It can be seen that healthy cases are scattered throughout the whole range of ESP and LFS, which suggests that no significant relationship exists between EF and ESP/LFS (Fig. 3.6c–f). Usually, higher contraction is associated with a higher pressure build-up (i.e. EF is usually associated with higher ESP), which is also suggested by the maximum and minimum ESP values being respectively placed in high and low EF (Fig. 3.6a,b). However, one may see that there exist “healthy” cases with either low or high ESP (Fig. 3.6c, d).

## 4 Discussion

In this study, we present a detailed description and a sensitivity analysis of a biophysically-detailed,bidirectionally-coupled ventricular electromechanical modelling and simulation framework including human-based electrophysiology, excitation–contraction coupling and active tension models. The computational model includes the four main components of a human ventricular electromechanical model: (1) solid mechanics, whose passive behaviour is based on the quasi-incompressible version of the Holzapfel and Ogden model [34], (2) the monodomain model for electrical propagation, (3) human ventricular cell electrophysiology using the modified version of the ORd model from [21], and (4) human active tension model as in [9], coupled to solid mechanics through an active stress framework. Two key mechanisms of mechano-electric feedback are also incorporated including how tissue deformation affects electrical diffusion, and the stretch-activated ionic currents. The main novelties and improvements of our framework, compared to previous published work, can be summarised as follows. We have focused on an integrated approach that couples state of the art biophysically-detailed models of electrophysiology and contractility, usually investigated separately or with simplified formulations [24], [25]. We adopted a dynamic formulation taking into account inertial forces, as it has been shown their relevance in cardiac electromechanics [22]. We implemented a pre-stress model to achieve diastolic pressure, allowing the simulation of the four phases of the cardiac cycle, together with an appropriate representation of epicardial mechanical boundary conditions.

From the simulated pressure–volume relationship which recapitulates the different phases of the cardiac cycle, physiologically-relevant mechanical biomarkers (EF, ESP, LFS, and WT) were extracted in a total of 200 simulations (i.e. 100 for one-at-a-time and 100 for LHS-based approaches). Simulation results using LHS were then used to compute the global sensitivities of relevant mechanical parameters, which have not been rigorously studied in human before. While recent studies [29] have addressed how electrophysiological biomarkers are affected by the incorporation of mechanics into a model of cardiac electrical diffusion under the presence of parameter uncertainty, so far the analysis has been restricted to idealised three-dimensional bars. This has implied that physiologically relevant mechanical biomarkers such as EF could not be assessed. Further comparisons will need to be performed once a more abundant body of other studies are available.

Our sensitivity analysis of model parameters with respect to mechanical biomarkers computed with an ellipsoidal model of the left ventricle of clinically-relevant volume [33] has shown that simulations with the human model and parameter variations yield values for the mechanical biomarkers ranging from healthy to disease scenarios. Varying a single model parameter in the simulations yielded variations in mechanical biomarkers that were nonlinear but monotonic (Fig. 3.2). This justified the use of LHS sampling-based PRCC for the evaluation of sensitivities. It is important to point out the strong influence of $T_{\text{ref}}$ on EF, which is attributable to the fact that higher $T_{\text{ref}}$ leads to higher active tension, causing higher contraction of the ventricles and thus higher EF. In fact, by looking at Fig. 3.2, one can see that variation of $T_{\text{ref}}$ alone yields physiological values of EF. Force production in a cardiac electromechanical model is mainly regulated by the active tension model, which needs a calcium input provided by the cell electrophysiology model; the human-based active tension model used in this study [9] was originally parametrised using very high values of calcium (coming from [77] instead of from a cell electrophysiology model, such as [6], [78]), but the values of calcium provided by [6] are much lower, which is translated into a decrease in force output. Therefore, scaling the active tension up to 7- or 8-fold was needed in order to reach physiological values of EF. An alternative would have been to re-parametrise the coupled cell electrophysiology and active tension model in a previous step; however, a simpler yet as effective approach is scaling $T_{\text{ref}}$, which is that considered in this study. This highlights the importance of calibrating the active tension model to a physiological healthy calcium transient, particularly when coupling it to the cell electrophysiology model.

When considering a global sensitivity analysis approach using LHS, a wide range of variability in mechanical biomarkers was obtained, including values lying on healthy and on diseased ranges (Fig. 3.4). Some of the LHS cases were also lying on the healthy ranges for all of the considered biomarkers. This means that such combinations of model parameters could be used as the baseline values for future studies involving this electromechanical model. The comparison with clinical ranges yielded some important observations. The simulated WT values obtained in this study lie on the lower physiological range (Fig. 3.4), which could potentially be due to the following reasons. The formulation of the computational model may need to be updated to include an active strain formulation with orthotropic activation as argued by [23], [79], *in lieu* of the active stress formulation used in this study, possibly even with heterogeneous orthotropic activation across the cardiac wall [23]. In our simulations, sheet directions are constant across the cardiac wall, with a sheetlet angle of 0 deg (i.e. parallel to the direction normal to the endocardial/epicardial surfaces). It has been suggested that while fibre angles vary linearly throughout the cardiac wall, sheetlet angles exhibit a non-monotonic variation, with the following approximate behaviour: going from a certain value x at the epicardium, to −x in the middle of the cardiac wall, to x again at the endocardium [80]. Sheetlet angles different than zero might also be needed as results may also depend on their distribution [27], [81]. Furthermore, possibly higher values of $k_{\text{ort}\;2}$ alone are needed to achieve higher, more physiological values of WT, which were not used in this study as it could compromise the positive-definiteness of the tangent operator of the Newton–Raphson scheme (the preconditioned conjugate gradients solver used for the linear algebraic systems resulting from the spatial discretisation of the solid mechanics subsystem did not converge for some cases with high $k_{\text{ort}\;2}$, even when using very small time steps). From a purely geometrical point of view, the thickness of the considered ventricle was 1.2 cm, which is on the upper range of human left ventricular wall thickness [82]; this may have also had an impact on WT values.

With respect to the sensitivities resulting from the global approach, the LHS-based approach yields tendencies in the sensitivities similar to those in the one-at-a-time results, in the sense that they generally have the same correlation signs. The PRCC suggest that EF, ESP and LFS are mainly dominated by one or two parameters each: EF is dominated by $T_{\text{ref}}$/$k_{\text{ort}\;2}$, ESP is dominated by $P_{\text{ej}}$/$k_{\text{ort}\;2}$, and LFS is dominated by ϕ/$k_{\text{ort}\;2}$ (Fig. 3.5). This means that extra effort could be placed into further experimentally characterising these parameters in order to obtain more accurate probability distributions of the considered mechanical biomarkers. On the contrary, WT does not seem to be clearly dominated by any of the considered input parameters, which suggests that it is likely affected by many different factors, such as the distribution of sheet and fibre directions, numerical formulation of contraction (i.e. active stress or active strain), or the parameter(s) that control the maximum level of contraction, among others. The distributions of EF, ESP and LFS with respect to their most influential parameters according to the PRCC values in Fig. 3.5 are seen in Fig. 3.6. One can see the shown correlations in the plots. Furthermore, the classification of cases according to their EF values (into “diseased” and “healthy”) helps in seeing that there is no direct correlation between EF and any of the other mechanical biomarkers, which means that these need to be assessed on a case-by-case basis instead of be inferred from EF values.

Some limitations of this study are worth remarking. The present study includes a sensitivity analysis of the effect of key parameters on mechanical biomarkers, but not all parameters are considered to enable study feasibility. The results can inform the design of further studies focused on geometry, mathematical formulation or boundary conditions, as these can also be important. An active stress formulation was used mainly because it is less invasive than an active strain formulation, and also because most active tension models are tailored to output active stress in the fibre direction, possibly due to experimental evidence at the cell scale [9], [83]. Our results show that simulations do not reproduce the whole range of WT reported in clinical studies. A simplified method was used, in which the thickness was calculated from two points that were initially found at the same height with respect to the base. Needless to say, during contraction this relative position is likely to change and thus introduce some bias in the thickness calculation. Additionally, experimental calculation of WT significantly depends on the evaluation method, with linear methods introducing error because of MRI slice positioning. If individual variation is considered, it can be seen that there is a substantial sparsity in results; for example, evaluation of WT on 9 healthy subjects led to values ranging from 18% to 100% [67]. It is likely that WT values are affected by the choice of volumetric term in the passive model for cardiac tissue and thus further research in this respect is warranted [84], [85]. The initiation of the system was done at the cellular level, and then an initiation phase was used to bring the geometry to an end-diastolic state; however, it is likely that the geometry also needs to be brought to periodic steady-state by repeating a cardiac cycle a few times. Needless to say, performing such thorough initiation needs huge computational resources and would indeed limit the number of LHS samples that could be performed. The geometry used in this study is an ellipsoidal left ventricular geometry, and therefore does not represent a biventricular geometry. Future studies can evaluate the role of anatomical variability in mechanical properties. In terms of the mechano-electric feedback, [86] suggested that stretch-dependent changes in capacitance result in changes in conduction velocity, and are important in order to retrieve an appropriate slow-down in conduction (such as that observed in rabbit ventricles [87]). In our study, we incorporate strain-dependent conductivity tensors as the main mechano-electric feedback mechanism, which is comparable in formulation to that of stretch-dependent space constants, and indeed allowed for conduction slow-down, as explained in Section 2.3.2.

## Appendix A Notation

Tensorial operations are denoted as follows. Single contraction of tensorial entities may appear as a⋅b ($a_{i}b_{i}$), a⋅B ($a_{i}B_{ij}$), Ab ($A_{ij}b_{j}$) or AB ($A_{ik}B_{kj}$), and we note that the scalar product symbol (⋅) only appears when the first entity to be contracted is a first-order tensor; double contraction of tensorial entities may appear as A:B ($A_{ij}B_{ij}$), A:B ($A_{ijkl}B_{kl}$), or A:B ($A_{ij}B_{ijkl}$). Other tensor products include a⊗b ($a_{i}b_{j}$), A⊗B ($A_{ij}B_{kl}$), $A\;\underline{⊗}\;B$ ($A_{ik}B_{jl}$), and $A\;\bar{⊗}\;B$ ($A_{il}B_{jk}$). Differential operators are denoted as follows. Divergence of the vector field a with respect to the reference and spatial configurations yields a scalar field, and are respectively denoted by $b=\nabla_{X}\cdot a\left(b=\frac{\partial a_{i}}{\partial X_{i}}\right)$ and $b=\nabla_{x}\cdot a\left(b=\frac{\partial a_{i}}{\partial x_{i}}\right)$. Divergence of the second-order tensor field A with respect to the reference and spatial configurations yields a vector field, and are respectively denoted by $b=\nabla_{X}\cdot A\left(b_{i}=\frac{\partial A_{ij}}{\partial X_{j}}\right)$ and $b=\nabla_{x}\cdot A\left(b_{i}=\frac{\partial A_{ij}}{\partial x_{j}}\right)$. Gradient of the scalar field a with respect to the reference and spatial configurations yields a vector field, and are respectively denoted by $b=\nabla_{X}a\left(b_{i}=\frac{\partial a}{\partial X_{i}}\right)$ and $b=\nabla_{x}a\left(b_{i}=\frac{\partial a}{\partial x_{i}}\right)$. Gradient of the vector field a with respect to the reference and spatial configurations yields a second-order tensor field, and is respectively denoted by $B=\nabla_{X}a\left(B_{ij}=\frac{\partial a_{i}}{\partial X_{j}}\right)$ and $B=\nabla_{x}a\left(B_{ij}=\frac{\partial a_{i}}{\partial x_{j}}\right)$. Gradient of the second-order tensor field A with respect to the second-order tensor field B yields a fourth-order tensor field, and is denoted by $\mathbb{C}=\frac{\partial A}{\partial B}\left({\mathbb{C}}_{ijkl}=\frac{\partial A_{ij}}{\partial B_{kl}}\right)$. Any other operations not specified here are described in the text in index notation whenever needed.

## Appendix B Cellular strong coupling

The coupling of the calcium subsystem and the excitation-contraction coupling model is obtained as follows. The total calcium concentration is defined as the sum of the free calcium plus calcium bound to both calmodulin and troponin [6], such that

$${\left[{\text{Ca}}^{2+}\right]}_{\text{TOTAL}}={\left[{\text{Ca}}^{2+}\right]}_{\text{i}}+{\left[{\text{Ca}}^{2+}\right]}_{\text{CMDN}}+{\left[{\text{Ca}}^{2+}\right]}_{\text{TRPN}}.$$

If algebraic formulations of the calmodulin and troponin buffers are considered, a time differentiation of the previous equations yields

$$\frac{\text{d}{\left[{\text{Ca}}^{2+}\right]}_{\text{TOTAL}}}{\text{d}t}=\frac{\text{d}{\left[{\text{Ca}}^{2+}\right]}_{\text{i}}}{\text{d}t}+\frac{\text{d}{\left[{\text{Ca}}^{2+}\right]}_{\text{CMDN}}}{\text{d}t}+\frac{\text{d}{\left[{\text{Ca}}^{2+}\right]}_{\text{TRPN}}}{\text{d}t}=\frac{\text{d}{\left[{\text{Ca}}^{2+}\right]}_{\text{i}}}{\text{d}t}+\frac{\frac{\text{d}{\left[{\text{Ca}}^{2+}\right]}_{\text{i}}}{\text{d}t}\left[\bar{\text{CMDN}}\right]\left({\left[{\text{Ca}}^{2+}\right]}_{\text{i}}+K_{\text{CMDN}}\right)-\frac{\text{d}{\left[{\text{Ca}}^{2+}\right]}_{\text{i}}}{\text{d}t}{\left[{\text{Ca}}^{2+}\right]}_{\text{i}}\left[\bar{\text{CMDN}}\right]}{{\left({\left[{\text{Ca}}^{2+}\right]}_{\text{i}}+K_{\text{CMDN}}\right)}^{2}}+\;\frac{\frac{\text{d}{\left[{\text{Ca}}^{2+}\right]}_{\text{i}}}{\text{d}t}\left[\bar{\text{TRPN}}\right]\left({\left[{\text{Ca}}^{2+}\right]}_{\text{i}}+K_{\text{TRPN}}\right)-\frac{\text{d}{\left[{\text{Ca}}^{2+}\right]}_{\text{i}}}{\text{d}t}{\left[{\text{Ca}}^{2+}\right]}_{\text{i}}\left[\bar{\text{TRPN}}\right]}{{\left({\left[{\text{Ca}}^{2+}\right]}_{\text{i}}+K_{\text{TRPN}}\right)}^{2}}=\frac{\text{d}{\left[{\text{Ca}}^{2+}\right]}_{\text{i}}}{\text{d}t}\left(1+\frac{\left[\bar{\text{CMDN}}\right]K_{\text{CMDN}}}{{\left({\left[{\text{Ca}}^{2+}\right]}_{\text{i}}+K_{\text{CMDN}}\right)}^{2}}+\frac{\left[\bar{\text{TRPN}}\right]K_{\text{TRPN}}}{{\left({\left[{\text{Ca}}^{2+}\right]}_{\text{i}}+K_{\text{TRPN}}\right)}^{2}}\right).$$

The total calcium transient is defined as

$$\frac{\text{d}{\left[{\text{Ca}}^{2+}\right]}_{\text{TOTAL}}}{\text{d}t}=-\left({\text{I}}_{\text{pCa}}+{\text{I}}_{\text{Cab}}-2{\text{I}}_{\text{NaCa}}\right)\frac{{\text{A}}_{\text{cap}}}{2\;\text{F}\;{\text{v}}_{\text{myo}}}-\text{J}\frac{{\text{v}}_{\text{nsr}}}{{\text{v}}_{\text{myo}}}+{\text{J}}_{\text{Ca}}\frac{{\text{v}}_{\text{ss}}}{{\text{v}}_{\text{myo}}},$$

and therefore

$$\frac{\text{d}{\left[{\text{Ca}}^{2+}\right]}_{\text{i}}}{\text{d}t}=\frac{1}{1+\frac{\left[\bar{\text{CMDN}}\right]K_{\text{CMDN}}}{{\left({\left[{\text{Ca}}^{2+}\right]}_{\text{i}}+K_{\text{CMDN}}\right)}^{2}}+\frac{\left[\bar{\text{TRPN}}\right]K_{\text{TRPN}}}{{\left({\left[{\text{Ca}}^{2+}\right]}_{\text{i}}+K_{\text{TRPN}}\right)}^{2}}}\left[\left(-{\text{I}}_{\text{pCa}}+{\text{I}}_{\text{Cab}}-2\;{\text{I}}_{\text{NaCa}}\right)\frac{{\text{A}}_{\text{cap}}}{2\;\text{F}\;{\text{v}}_{\text{myo}}}-\text{J}\frac{{\text{v}}_{\text{nsr}}}{{\text{v}}_{\text{myo}}}+{\text{J}}_{\text{Ca}}\frac{{\text{v}}_{\text{ss}}}{{\text{v}}_{\text{myo}}}\right].$$

Another possibility is to leave the transient of calcium bound to troponin as an unknown [88], which would lead to

$$\frac{\text{d}{\left[{\text{Ca}}^{2+}\right]}_{\text{i}}}{\text{d}t}=\frac{1}{1+\frac{\left[\bar{\text{CMDN}}\right]K_{\text{CMDN}}}{{\left({\left[{\text{Ca}}^{2+}\right]}_{\text{i}}+K_{\text{CMDN}}\right)}^{2}}}[-\left({\text{I}}_{\text{pCa}}+{\text{I}}_{\text{Cab}}-2\;{\text{I}}_{\text{NaCa}}\right)\frac{{\text{A}}_{\text{cap}}}{2\;\text{F}\;{\text{v}}_{\text{myo}}}-\text{J}\frac{{\text{v}}_{\text{nsr}}}{{\text{v}}_{\text{myo}}}+\;{\text{J}}_{\text{Ca}}\frac{{\text{v}}_{\text{ss}}}{{\text{v}}_{\text{myo}}}-\frac{\text{d}{\left[{\text{Ca}}^{2+}\right]}_{\text{TRPN}}}{\text{d}t}].$$

The transient of calcium bound to troponin can be then defined as the transient of fraction of troponin C units with calcium bound to its regulatory binding site multiplied by a constant maximum concentration of calcium ions that can bind to troponin (${\left[\text{TRPN}\right]}_{\text{max}}=0.07\;\text{mM}$), leading to

$$\frac{\text{d}{\left[{\text{Ca}}^{2+}\right]}_{\text{TRPN}}}{\text{d}t}=0.07\;\frac{\text{dCaTRPN}}{\text{d}t},\;\text{CaTRPN}\in \left\{q\right\}.$$

## Appendix C Finite element discretisation

The discretisation in space is carried out by means of a conforming finite element scheme. A Galerkin method for (2.17) consists in choosing finite-dimensional subspaces of the trial and tests spaces introduced in Section Section 2.4, associated with a regular partition $T_{h}$ of $\Omega_{0}$ into tetrahedra K of maximum diameter $h_{K}$, and define the meshsize as $h≔\max\left\{h_{K}:K\in T_{h}\right\}$. In this case piecewise linear and overall continuous polynomials for displacements and transmembrane potential are used; that is, the conforming finite element subspaces are (C.1)

$$H^{h}=\left\{v^{h}\in C^{0}\left(\bar{\Omega_{0}}\right):v^{h}{|}_{K}\in P_{1}{\left(K\right)}^{3},\forall K\in T_{h}\right\},$$

$$V^{h}=\left\{\varphi^{h}\in C^{0}\left(\bar{\Omega_{0}}\right):\varphi^{h}{|}_{K}\in P_{1}\left(K\right),\forall K\in T_{h}\right\},$$

where $P_{r}\left(K\right)$ denotes the space of polynomial functions of degree s≤r defined locally on the element K. For example, each discrete function (e.g. a component of the displacement or an approximation of the transmembrane potential), say v, at a given time t is written as

(C.2) $$v^{h}\left(X,t\right)=\overset{\text{dof}}{\underset{i}{\sum }}v_{i}\left(t\right)\psi_{i}\left(X\right),$$

where $\psi_{i}$ denotes continuous piecewise polynomial nodal basis functions and $v_{i}$ are the nodal values of the generic finite element function.

The natural choice for a finite element approximation of Q is the space of element-wise constant functions $Q^{h}=\left\{r^{h}\in L^{2}\left(\Omega_{0}\right):r^{h}{|}_{K}\in P_{0}\left(K\right),\forall K\in T_{h}\right\}$. The absence of spatial gradients in (2.17e), (2.17c) and (2.17d), suggests that their discretisation can be carried out separately from the coupled system and performed element by element. However, due to the large dimension of $\left\{w^{h},c^{h}\right\}$ (41 state variables), and since typical tetrahedral meshes possess a considerably smaller number of nodes than elements, the subproblems associated with $w^{h}$ and $c^{h}$ can be done nodally [89]. On the other hand, the system for $q^{h}$ is solved locally at the quadrature points, as this may contribute to improved stability, avoiding additional interpolation errors related to the dependence on the gradients of $u^{h}$ [26], [90].

## Appendix D Time discretisation and operator splitting

A partition of the time interval (0,T] into $N_{t}$ subintervals of length Δt is considered. Time advancement of the nonlinear coupled problem is separated between the monodomain equations and nonlinear mechanics by using a classical first-order operator-splitting scheme which, together with the appropriate time differentiation schemes, here Newmark-β for second-order derivatives and backward Euler for first-order derivatives, results in the following fully-discrete systems:

Start from known values of the $L^{2}$-projections of the initial displacement and transmembrane potential $u_{-1}^{h}=u_{0}^{h}$, $u_{0}^{h}$, $V_{0}^{h}$, as well as for known values of $q_{0}^{i}$ at the quadrature points i and known values of $\left\{w_{0}^{j},c_{0}^{j}\right\}$ at the degrees of freedom j. Then, for each $n=0,1,\ldots ,N_{t}+1$, perform the sequential iteration

*Step 1:* knowing $F_{n}^{h}$, ${\overset{ˆ}{q}}_{n}^{h}$, $V_{n}^{h},w_{n}^{h},c_{n}^{h}$, find $V_{n+1}^{h},w_{n+1}^{h},c_{n+1}^{h}$ such that

$$\chi C_{\text{m}}\int_{\Omega_{0}}\;J_{n}^{h}\frac{V_{n+1}^{h}-V_{n}^{h}}{\Delta t}\varphi^{h}\;\text{d}\Omega_{0}+\int_{\Omega_{0}}J_{n}^{h}{\left(F_{n}^{h}\right)}^{-1}D{\left(F_{n}^{h}\right)}^{-\text{T}}\nabla_{X}V_{n+1}^{h}\cdot \nabla_{X}\varphi^{h}\;\text{d}\Omega_{0}$$

$$\;+\chi \int_{\Omega_{0}}J_{n}^{h}I_{\text{ion}\;n+1}^{h}\varphi^{h}\;\text{d}\Omega_{0}+\chi \int_{\Omega_{0}}J_{n}^{h}\left[I_{\text{SAC}\;n+1}^{h}-I_{\text{app}\;n+1}\right]\varphi^{h}\;\text{d}\Omega_{0}=0,\;\forall \varphi^{h}\in V^{h},$$

 coupled with nodal equations that, owing to (C.1)–(C.2), can be equivalently written as

$$\frac{1}{\Delta t}w_{n+1}^{h}=\frac{1}{\Delta t}w_{n}^{h}+m_{w}\left(V_{n+1}^{h},w_{n+1}^{h},c_{n+1}^{h}\right),$$

$$\frac{1}{\Delta t}c_{n+1}^{h}=\frac{1}{\Delta t}c_{n}^{h}+m_{c}\left(V_{n+1}^{h},w_{n+1}^{h},c_{n+1}^{h},q_{n+1}^{h}\right).$$

 Here ${\overset{ˆ}{q}}_{n}^{h}$ denote values of the active tension model state variables (solved at the integration points in Step 2, below) interpolated at the nodes. The nonlinear problem in Step 1 is further split into the two following components by an additional first-order operator splitting scheme applied to separate the reaction from the diffusion terms in the monodomain equation (2.17b), leading to

*Step 1.1:* knowing ${\overset{ˆ}{q}}_{n}^{h}$, $V_{n}^{h},w_{n}^{h},c_{n}^{h}$ find the nodal values of $V_{⋆}^{h},w_{n+1}^{h},c_{n+1}^{h}$ from the nodal evaluations (D.1)

$$\frac{w_{n+1}^{h}-w_{n}^{h}}{\Delta t}-m_{w}\left(V_{⋆}^{h},w_{†}^{h},c_{n}^{h}\right)=0,$$

$$\frac{c_{n+1}^{h}-c_{n}^{h}}{\Delta t}-m_{c}\left(V_{⋆}^{h},w_{n}^{h},c_{†}^{h},{\overset{ˆ}{q}}_{n}^{h}\right)=0,$$

$$C_{\text{m}}\frac{V_{⋆}^{h}-V_{n}^{h}}{\Delta t}=-I_{\text{ion}}\left(V_{⋆}^{h},w_{n+1}^{h},c_{n+1}^{h},{\overset{ˆ}{q}}_{n}^{h}\right)-I_{\text{SAC}\;n+1}+I_{\text{app}\;n+1}.$$

*Step 1.2:* knowing $F_{n}^{h}$, find $V_{n+1}^{h}\in V^{h}$ such that (D.2)

$$\chi C_{\text{m}}\int_{\Omega_{0}}J_{n}^{h}\frac{V_{n+1}^{h}-V_{⋆}^{h}}{\Delta t}\varphi^{h}\;\text{d}\Omega_{0}+\int_{\Omega_{0}}J_{n}^{h}{\left(F_{n}^{h}\right)}^{-1}D{\left(F_{n}^{h}\right)}^{-\text{T}}\nabla_{X}V_{n+1}^{h}\cdot \nabla_{X}\varphi^{h}\;\text{d}\Omega_{0}=0,\;\forall \varphi^{h}\in V^{h}.$$

The subscript ⋆ denotes the transmembrane potential computed after Step 1.1 and the subscript † indicates that the nonlinear contributions in the reaction terms associated with the gating variables and ionic concentrations in (D.1) are solved explicitly, except for the linear contributions. Therefore, Step 1.1 is subdivided into time substeps in order to satisfy stability conditions for ODE explicit integration schemes.

*Step 2:* knowing $c_{n+1}^{h}$ from Step 1, as well as $q_{n}^{h},u_{n}^{h},u_{n-1}^{h}$, find $u_{n+1}^{h}\in H^{h}$ and $q_{n+1}^{h}\in Q^{n_{q}}$ such that

(D.3) $$\int_{\Omega_{0}}\rho_{0}\frac{4}{\Delta t^{2}}\left(u_{n+1}^{h}-u_{n}^{h}\right)\cdot v^{h}\;\text{d}\Omega_{0}+\int_{\Omega_{0}}F_{n+1}^{h}S_{n+1}^{h}:\nabla_{X}v^{h}\;\text{d}\Omega_{0}-\int_{\partial \Omega_{0\;\text{epi}}}\left(k_{\text{epi}}u_{n+1}^{h}\cdot n_{0}\right)n_{0}\cdot v^{h}\;\text{d}\partial \Omega_{0}\;=\int_{\Omega_{0}}\;\rho_{0}\left(\frac{4}{\Delta t^{2}}y_{n}^{h}+z_{n}^{h}\right)\cdot v^{h}\;\text{d}\Omega_{0}+\int_{\partial \Omega_{0\;\text{endo}}}\;\;\;\;\;J_{n+1}^{h}P_{\text{endo}\;n+1}{\left(F_{n+1}^{h}\right)}^{-\text{T}}\;\;n_{0}\cdot v^{h}\;\text{d}\partial \Omega_{0},\;\forall v^{h}\in H^{h},$$

with

$$y_{n}^{h}=\frac{2}{\Delta t}\left(u_{n}^{h}-u_{n-1}^{h}\right)-y_{n-1}^{h},$$

$$z_{n}^{h}=\frac{4}{\Delta t^{2}}\left(u_{n}^{h}-u_{n-1}^{h}\right)-\frac{4}{\Delta t}y_{n-1}^{h}-z_{n-1}^{h},$$

 coupled to a quadrature point-wise evolution equation for the active tension model state variables, solved implicitly as in [26], [46]

(D.4) $$\frac{1}{\Delta t}q_{n+1}^{h}=\frac{1}{\Delta t}q_{n}^{h}+m_{q}\left(q_{n+1}^{h},{\overset{\sim }{c}}_{n+1}^{h},\lambda_{\text{f}\;n+1}^{h},\frac{\lambda_{\text{f}\;n+1}^{h}-\lambda_{\text{f}\;n}^{h}}{\Delta t}\right),$$

and coupled to the algebraic equation for the active tension (2.9), and the following implicit evaluations, also performed at the Gauss integration points ($P_{\text{endo}}$ is evaluated at the Gauss integration points of the corresponding boundary, instead of volume, elements),

$$\frac{1}{\Delta t}\sigma_{0\;n+1}=\frac{1}{\Delta t}\sigma_{0\;n}+\left\{\begin{array}{cc} \frac{1}{C_{\text{p},\text{endo}\;n+1}}\frac{V_{\text{endo}\;n+1}-{\text{EDV}}_{\text{endo}}}{\Delta t}+\frac{1}{C_{\text{v,endo}}}\frac{V_{\text{endo}\;n+1}-V_{\text{endo}\;n}}{\Delta t^{2}}\;\; & \text{in}\;\text{initiation}\\ 0\;\; & \text{otherwise} \end{array}\right),$$

$$\frac{P_{\text{endo}\;n+1}}{\Delta t}=\frac{P_{\text{endo}\;n}}{\Delta t}+\left\{\begin{array}{cc} \frac{P_{\text{endo},0}\frac{t_{n}+\Delta t}{T_{0}}-P_{\text{endo}\;n}}{\Delta t}\;\; & \text{in}\;\text{initiation}\\ -\left(\frac{1}{C_{\text{p}\;n+1}}\frac{V_{\text{endo}\;n+1}-{\text{EDV}}_{\text{endo}}}{\Delta t}+\frac{1}{C_{\text{v}}}\frac{V_{\text{endo}\;n+1}-V_{\text{endo}\;n}}{\Delta t^{2}}\right)\;\; & \text{in isovolumetric contraction}\\ -\left(\frac{1}{C_{\text{art}}}\frac{V_{\text{endo}\;n+1}-V_{\text{endo}\;n}}{\Delta t}+\frac{P_{\text{art}\;n+1}}{R_{\text{art}}}\right)\;\; & \text{in ejection}\\ -\left(\frac{1}{C_{\text{p}\;n+1}}\frac{V_{\text{endo}\;n+1}-{\text{ESV}}_{\text{endo}}}{\Delta t}+\frac{1}{C_{\text{v}}}\frac{V_{\text{endo}\;n+1}-V_{\text{endo}\;n}}{\Delta t^{2}}\right)\;\; & \text{in isovolumetric relaxation}\\ -\gamma \frac{V_{\text{endo}\;n+1}-V_{\text{endo}\;n}}{\Delta t}\;\; & \text{otherwise} \end{array}\right)$$

 where EDV is the end-diastolic volume. In (D.4), the quantities ${\overset{\sim }{c}}_{n+1}^{h}$ are values of the cell electrophysiology state variables solved at the nodes in Step 1.1, and then evaluated at the integration points.

## Appendix E Linearisation

After applying the aforementioned time discretisation and operator splitting schemes the sequential problems (D.1), (D.2) involved in Step 1 become linear while the two sequential problems in Step 2 (D.3), (D.4) remain nonlinear. Therefore, two separate Newton–Raphson linearisation schemes are considered for the main stage of Newmark-β’s method and for the computation of the active tension model state variables.

For (D.3), knowing $u_{k=0}^{h}=u_{n}^{h}$, and for $k=0,1,\ldots ,n_{\text{maxNR}}$, or until convergence, one seeks displacement increments $\delta u_{k+1}^{h}=u_{k+1}^{h}-u_{k}^{h}$ such that (time subscripts n+1, but not n, are dropped from now onwards; Newton–Raphson iteration subscripts k or k+1 are used instead)

(E.1) $$\int_{\Omega_{0}}\frac{4\rho_{0}}{\Delta t^{2}}\;v^{h}\cdot \delta u_{k+1}^{h}\;\text{d}\Omega_{0}+\int_{\Omega_{0}}\nabla_{X}v^{h}:{\frac{\partial F^{h}S^{h}}{\partial F^{h}}|}_{u_{k}^{h}}:\left({\frac{\partial F^{h}}{\partial u^{h}}|}_{u_{k}^{h}}\cdot \delta u_{k+1}^{h}\right)\;\text{d}\Omega_{0}\;-k_{\text{epi}}\int_{\partial \Omega_{0\;\text{epi}}}v^{h}\cdot \left(n_{0}⊗n_{0}\right)\delta u_{k+1}^{h}\text{d}\partial \Omega_{0}-\int_{\partial \Omega_{0\;\text{endo}}}P_{\text{endo}\;k}\left[v^{h}\cdot {\left(F_{k}^{h}\right)}^{-\text{T}}n_{0}\right]{\frac{\partial J^{h}}{\partial u^{h}}|}_{u_{k}^{h}}\cdot \delta u_{k+1}^{h}\;\text{d}\partial \Omega_{0}\;-\int_{\partial \Omega_{0\;\text{endo}}}J_{k}^{h}\left[v^{h}\cdot {\left(F_{k}^{h}\right)}^{-\text{T}}n_{0}\right]{\frac{\partial P_{\text{endo}}}{\partial u^{h}}|}_{u_{k}^{h}}\cdot \delta u_{k+1}^{h}\;\text{d}\partial \Omega_{0}\;-\int_{\partial \Omega_{0\;\text{endo}}}J_{k}^{h}P_{\text{endo}\;k}\;v^{h}\cdot {\frac{\partial {\left(F^{h}\right)}^{-\text{T}}}{\partial u^{h}}|}_{u_{k}^{h}}:\left(n_{0}⊗\delta u_{k+1}^{h}\right)\;\text{d}\partial \Omega_{0}$$

$$=\int_{\Omega_{0}}\;\rho_{0}\left(\frac{4}{\Delta t^{2}}\left[y_{n}^{h}-u_{k}^{h}\right]+z_{n}^{h}\right)\cdot v^{h}\;\text{d}\Omega_{0}-\int_{\Omega_{0}}F_{k}^{h}S_{k}^{h}:\nabla_{X}v^{h}\;\text{d}\partial \Omega_{0}\;\;+\int_{\partial \Omega_{0\;\text{endo}}}J_{k}^{h}P_{\text{endo}\;k}{\left(F_{k}^{h}\right)}^{-\text{T}}n_{0}\cdot v^{h}\;\text{d}\partial \Omega_{0}+k_{\text{epi}}\int_{\partial \Omega_{0\;\text{epi}}}J_{k}^{h}\left(u_{k}^{h}\cdot n_{0}\right)n_{0}\cdot v^{h}\;\text{d}\partial \Omega_{0},\;\forall v^{h}\in H^{h},$$

 where the Fréchet derivatives needed in (E.1) are

$$\frac{\partial u^{h}}{\partial u^{h}}=I_{u^{h}},\;\frac{\partial F^{h}S^{h}}{\partial F^{h}}=I\;\underline{⊗}\;S^{h}+2F^{h}\frac{\partial S^{h}}{\partial C^{h}}{\left(F^{h}\right)}^{\text{T}},\;\frac{\partial J^{h}}{\partial u^{h}}=J^{h}{\left(F^{h}\right)}^{\text{-T}}\frac{\partial F^{h}}{\partial u^{h}}.$$

Here $I_{u^{h}}$ is the identity operator of dimension equal to the number of displacement degrees of freedom, $\frac{\partial F^{h}}{\partial u^{h}}$ is the discrete gradient of the displacement nodal basis functions,

$$2F^{h}\frac{\partial S^{h}}{\partial C^{h}}{\left(F^{h}\right)}^{\text{T}}\equiv 2F_{im}^{h}\frac{\partial S_{mj}^{h}}{\partial C_{nl}^{h}}F_{kn}^{h},$$

$$J^{h}P_{\text{endo}}\;v^{h}\cdot {\frac{\partial {\left(F^{h}\right)}^{-\text{T}}}{\partial u^{h}}|}_{u_{k}^{h}}:\left(n_{0}⊗\delta u_{k+1}^{h}\right)\equiv -\frac{1}{2}J^{h}P_{\text{endo}}v_{i}\left(F_{jm}^{-1}F_{in}^{-1}+F_{jn}^{-1}F_{im}^{-1}\right)\frac{\partial F_{mn}}{\partial u_{k}}n_{j}\delta u_{k},$$

 (on the RHS of the two previous expressions the iteration subscripts k or k+1 have been dropped in favour of tensorial indices) and the precise definition of

$$\frac{\partial S^{h}}{\partial C^{h}}=\frac{\partial S_{\text{pas}}^{h}}{\partial C^{h}}+\frac{\partial S_{\text{act}}^{h}}{\partial C^{h}},$$

can be found in Appendix F. At the end of each Newton–Raphson iteration, the solution is updated and the iterations are terminated once the relative $\ell^{2}$-norm of the solution increment drops below a fixed tolerance of $10^{-5}$.

On the other hand, the updated values of the state variables in (D.4) are obtained from the Newton–Raphson iteration defined by

(E.2) $$q_{k+1}^{h}={\left(I_{6}-\Delta t{\frac{\partial m_{q}}{\partial q}|}_{q_{k}^{h},{\overset{\sim }{c}}_{n+1}^{h},\lambda_{\text{f}\;k}^{h},\frac{\lambda_{\text{f}\;k}^{h}-\lambda_{\text{f}\;n}^{h}}{\Delta t}}\right)}^{-1}\;\;\left(q_{k}^{h}-q_{n}^{h}-\Delta t\;{m{}_{q}|}_{q_{k}^{h},{\overset{\sim }{c}}_{n+1}^{h},\lambda_{\text{f}\;k}^{h},\frac{\lambda_{\text{f}\;k}^{h}-\lambda_{\text{f}\;n}^{h}}{\Delta t}}\right)+q_{k}^{h},$$

where $I_{6}$ is the identity operator of dimension 6, and the non-zero entries of the 6 × 6 Jacobian operator in (E.2) are defined as

$${\left(\frac{\partial m_{q}}{\partial q}\right)}_{11}=1-k_{\text{su}}-\gamma_{\text{su}},\;{\left(\frac{\partial m_{q}}{\partial q}\right)}_{12}=k_{\text{ws}},\;{\left(\frac{\partial m_{q}}{\partial q}\right)}_{15}=-S\frac{\partial \gamma_{\text{su}}}{\partial \zeta_{\text{s}}},\;{\left(\frac{\partial m_{q}}{\partial q}\right)}_{21}=-k_{\text{uw}},{\left(\frac{\partial m_{q}}{\partial q}\right)}_{22}=1-k_{\text{uw}}-k_{\text{wu}}-k_{\text{ws}}-\gamma_{\text{wu}},\;{\left(\frac{\partial m_{q}}{\partial q}\right)}_{24}=-k_{\text{uw}},\;{\left(\frac{\partial m_{q}}{\partial q}\right)}_{26}=-W\frac{\partial \gamma_{\text{wu}}}{\partial \zeta_{\text{w}}},{\left(\frac{\partial m_{q}}{\partial q}\right)}_{33}=1-k_{\text{TRPN}}\left[{\left(\frac{{\left[{\text{Ca}}^{2+}\right]}_{\text{i}}}{{\left[{\text{Ca}}^{2+}\right]}_{\text{T50}}}\right)}^{n_{\text{TRPN}}}+1\right],\;{\left(\frac{\partial m_{q}}{\partial q}\right)}_{41}=-K_{\text{u}}{\text{CaTRPN}}^{\frac{n_{\text{Tm}}}{2}},{\left(\frac{\partial m_{q}}{\partial q}\right)}_{42}=-K_{\text{u}}{\text{CaTRPN}}^{\frac{n_{\text{Tm}}}{2}},\;{\left(\frac{\partial m_{q}}{\partial q}\right)}_{43}=\frac{n_{\text{Tm}}}{2}{\text{CaTRPN}}^{\left(\frac{n_{\text{Tm}}}{2}-1\right)}\left[K_{\text{u}}\left(1-B-S-W\right)-k_{\text{u}}B\right],{\left(\frac{\partial m_{q}}{\partial q}\right)}_{44}=1-{\text{CaTRPN}}^{\frac{n_{\text{Tm}}}{2}}\left(K_{\text{u}}+k_{\text{u}}\right),\;{\left(\frac{\partial m_{q}}{\partial q}\right)}_{55}=1-c_{\text{s}},\;{\left(\frac{\partial m_{q}}{\partial q}\right)}_{66}=1-c_{\text{w}},$$

with $K_{\text{u}}=\frac{k_{\text{u}}}{1-r_{\text{s}}-\left(1-r_{\text{s}}\right)r_{\text{w}}}$.

## Appendix F Explicit form of the tangent stiffness tensor

The tangent modulus for the passive part of the Second Piola–Kirchhoff stress reads as

$${\mathbb{C}}_{\text{pas}}^{h}=2\frac{\partial S_{\text{pas}}^{h}}{\partial C^{h}}=2{\left(J^{h}\right)}^{-\frac{2}{3}}a\;{\text{e}}^{b\left({\bar{I}}_{1}^{h}-3\right)}\left(-\frac{1}{3}I⊗{\left(C^{h}\right)}^{-1}-\frac{1}{3}{\left(C^{h}\right)}^{-1}⊗I+\frac{1}{9}I_{1}^{h}{\left(C^{h}\right)}^{-1}⊗{\left(C^{h}\right)}^{-1}+\;\frac{1}{6}I_{1}^{h}\left[{\left(C^{h}\right)}^{-1}\underline{⊗}{\left(C^{h}\right)}^{-1}+{\left(C^{h}\right)}^{-1}\bar{⊗}{\left(C^{h}\right)}^{-1}\right]\right)+\;2{\left(J^{h}\right)}^{-\frac{4}{3}}a\;b\;{\text{e}}^{b\left({\bar{I}}_{1}^{h}-3\right)}\left[\left(I-\frac{1}{3}I_{1}^{h}{\left(C^{h}\right)}^{-1}\right)⊗\left(I-\frac{1}{3}I_{1}^{h}{\left(C^{h}\right)}^{-1}\right)\right]+\;4{\left(J^{h}\right)}^{-\frac{2}{3}}a_{\text{f}}{\left({\bar{I}}_{\text{4f}}^{h}-1\right)}_{+}{\text{e}}^{b_{\text{f}}{\left({\bar{I}}_{\text{4f}}^{h}-1\right)}_{+}^{2}}\left(-\frac{1}{3}f_{0}⊗f_{0}⊗{\left(C^{h}\right)}^{-1}-\frac{1}{3}{\left(C^{h}\right)}^{-1}⊗f_{0}⊗f_{0}+\;\frac{1}{9}I_{\text{4f}}^{h}{\left(C^{h}\right)}^{-1}⊗{\left(C^{h}\right)}^{-1}+\frac{1}{6}I_{\text{4f}}^{h}\left[{\left(C^{h}\right)}^{-1}\underline{⊗}{\left(C^{h}\right)}^{-1}+{\left(C^{h}\right)}^{-1}\bar{⊗}{\left(C^{h}\right)}^{-1}\right]\right)+\;4{\left(J^{h}\right)}^{-\frac{4}{3}}a_{\text{f}}\;{\text{e}}^{b_{\text{f}}{\left({\bar{I}}_{\text{4f}}^{h}-1\right)}_{+}^{2}}\left[\left(f_{0}⊗f_{0}-\frac{1}{3}I_{\text{4f}}^{h}{\left(C^{h}\right)}^{-1}\right)⊗\left(f_{0}⊗f_{0}-\frac{1}{3}I_{\text{4f}}^{h}{\left(C^{h}\right)}^{-1}\right)\right]+\;8{\left(J^{h}\right)}^{-\frac{4}{3}}a_{\text{f}}\;b_{\text{f}}{\left({\bar{I}}_{\text{4f}}^{h}-1\right)}_{+}^{2}{\text{e}}^{b_{\text{f}}{\left({\bar{I}}_{\text{4f}}^{h}-1\right)}_{+}^{2}}\left[\left(f_{0}⊗f_{0}-\frac{1}{3}I_{\text{4f}}^{h}{\left(C^{h}\right)}^{-1}\right)⊗\left(f_{0}⊗f_{0}-\frac{1}{3}I_{\text{4f}}^{h}{\left(C^{h}\right)}^{-1}\right)\right]+\;4{\left(J^{h}\right)}^{-\frac{2}{3}}a_{\text{s}}{\left({\bar{I}}_{\text{4s}}^{h}-1\right)}_{+}{\text{e}}^{b_{\text{s}}{\left({\bar{I}}_{\text{4s}}^{h}-1\right)}_{+}^{2}}\left(-\frac{1}{3}s_{0}⊗s_{0}⊗{\left(C^{h}\right)}^{-1}-\frac{1}{3}{\left(C^{h}\right)}^{-1}⊗s_{0}⊗s_{0}+\;\frac{1}{9}I_{\text{4s}}^{h}{\left(C^{h}\right)}^{-1}⊗{\left(C^{h}\right)}^{-1}+\frac{1}{6}I_{\text{4s}}^{h}\left[{\left(C^{h}\right)}^{-1}\underline{⊗}{\left(C^{h}\right)}^{-1}+{\left(C^{h}\right)}^{-1}\bar{⊗}{\left(C^{h}\right)}^{-1}\right]\right)+\;4{\left(J^{h}\right)}^{-\frac{4}{3}}a_{\text{s}}\;{\text{e}}^{b_{\text{s}}{\left({\bar{I}}_{\text{4s}}^{h}-1\right)}_{+}^{2}}\left[\left(s_{0}⊗s_{0}-\frac{1}{3}I_{\text{4s}}^{h}{\left(C^{h}\right)}^{-1}\right)⊗\left(s_{0}⊗s_{0}-\frac{1}{3}I_{\text{4s}}^{h}{\left(C^{h}\right)}^{-1}\right)\right]+\;8{\left(J^{h}\right)}^{-\frac{4}{3}}a_{\text{s}}\;b_{\text{s}}{\left({\bar{I}}_{\text{4s}}^{h}-1\right)}_{+}^{2}{\text{e}}^{b_{\text{s}}{\left({\bar{I}}_{\text{4s}}^{h}-1\right)}_{+}^{2}}\left[\left(s_{0}⊗s_{0}-\frac{1}{3}I_{\text{4s}}^{h}{\left(C^{h}\right)}^{-1}\right)⊗\left(s_{0}⊗s_{0}-\frac{1}{3}I_{\text{4s}}^{h}{\left(C^{h}\right)}^{-1}\right)\right]+\;4{\left(J^{h}\right)}^{-\frac{2}{3}}a_{\text{fs}}{\bar{I}}_{\text{8fs}}^{h}{\text{e}}^{b_{\text{fs}}{\left({\bar{I}}_{\text{8fs}}^{h}\right)}^{2}}\left(-\frac{1}{6}\left(f_{0}⊗s_{0}+s_{0}⊗f_{0}\right)⊗{\left(C^{h}\right)}^{-1}-\frac{1}{6}{\left(C^{h}\right)}^{-1}⊗\left(f_{0}⊗s_{0}+s_{0}⊗f_{0}\right)+\;\frac{1}{9}I_{\text{8fs}}^{h}{\left(C^{h}\right)}^{-1}⊗{\left(C^{h}\right)}^{-1}+\frac{1}{6}I_{\text{8fs}}^{h}\left[{\left(C^{h}\right)}^{-1}\underline{⊗}{\left(C^{h}\right)}^{-1}+{\left(C^{h}\right)}^{-1}\bar{⊗}{\left(C^{h}\right)}^{-1}\right]\right)+\;4{\left(J^{h}\right)}^{-\frac{4}{3}}a_{\text{fs}}\;{\text{e}}^{b_{\text{fs}}{\left({\bar{I}}_{\text{8fs}}^{h}\right)}^{2}}\left[\left(\frac{1}{2}\left(f_{0}⊗s_{0}+s_{0}⊗f_{0}\right)-\frac{1}{3}I_{\text{8fs}}^{h}{\left(C^{h}\right)}^{-1}\right)\right)\left(⊗\left(\frac{1}{2}\left(f_{0}⊗s_{0}+s_{0}⊗f_{0}\right)-\frac{1}{3}I_{\text{8fs}}^{h}{\left(C^{h}\right)}^{-1}\right)\right]+\;8{\left(J^{h}\right)}^{-\frac{4}{3}}a_{\text{fs}}\;b_{\text{fs}}{\left({\bar{I}}_{\text{8fs}}^{h}\right)}^{2}{\text{e}}^{b_{\text{fs}}{\left({\bar{I}}_{\text{8fs}}^{h}\right)}^{2}}\left[\left(\frac{1}{2}\left(f_{0}⊗s_{0}+s_{0}⊗f_{0}\right)-\frac{1}{3}I_{\text{8fs}}^{h}{\left(C^{h}\right)}^{-1}\right)\right)\left(⊗\left(\frac{1}{2}\left(f_{0}⊗s_{0}+s_{0}⊗f_{0}\right)-\frac{1}{3}I_{\text{8fs}}^{h}{\left(C^{h}\right)}^{-1}\right)\right]+\;2K\left(J^{h}\right)\left[\left(2J^{h}-1\right){\left(C^{h}\right)}^{-1}⊗{\left(C^{h}\right)}^{-1}-\frac{1}{2}\left(J^{h}-1\right)\left({\left(C^{h}\right)}^{-1}\underline{⊗}{\left(C^{h}\right)}^{-1}+{\left(C^{h}\right)}^{-1}\bar{⊗}{\left(C^{h}\right)}^{-1}\right)\right].$$

The consistent tangent modulus of the active part of the Second Piola–Kirchhoff stress tensor is defined as

$${\mathbb{C}}_{\text{act}}^{h}=2\frac{\partial S_{\text{act}}^{h}}{\partial C^{h}}=2\left[\frac{\partial \left(T_{\text{act}}^{h}+\sigma_{0}^{h}\right)}{\partial C^{h}}\frac{f_{0}⊗f_{0}}{{\left(\lambda_{\text{f}}^{h}\right)}^{2}}+\left(T_{\text{act}}^{h}+\sigma_{0}^{h}\right)\frac{\partial }{\partial C^{h}}\left(\frac{1}{{\left(\lambda_{\text{f}}^{h}\right)}^{2}}\right)f_{0}⊗f_{0}+\;k_{\text{ort}\;1}\frac{\partial T_{\text{act}}^{h}}{\partial C^{h}}\frac{s_{0}⊗s_{0}}{{\left(\lambda_{\text{s}}^{h}\right)}^{2}}+k_{\text{ort}\;1}T_{\text{act}}^{h}\frac{\partial }{\partial C^{h}}\left(\frac{1}{{\left(\lambda_{\text{s}}^{h}\right)}^{2}}\right)s_{0}⊗s_{0}+\;k_{\text{ort}\;2}\frac{\partial T_{\text{act}}^{h}}{\partial C^{h}}\frac{n_{0}⊗n_{0}}{{\left(\lambda_{\text{n}}^{h}\right)}^{2}}+k_{\text{ort}\;2}T_{\text{act}}^{h}\frac{\partial }{\partial C^{h}}\left(\frac{1}{{\left(\lambda_{\text{n}}^{h}\right)}^{2}}\right){\overset{¯}{n}}_{0}⊗{\overset{¯}{n}}_{0}\right],$$

where

$$\frac{\partial \lambda_{\text{f}}^{h}}{\partial C^{h}}=\frac{1}{2\lambda_{\text{f}}^{h}}f_{0}⊗f_{0},\;\frac{\partial \lambda_{\text{s}}^{h}}{\partial C^{h}}=\frac{1}{2\lambda_{\text{s}}^{h}}s_{0}⊗s_{0},\;\frac{\partial \lambda_{\text{n}}^{h}}{\partial C^{h}}=\frac{1}{2\lambda_{\text{n}}^{h}}{\overset{¯}{n}}_{0}⊗{\overset{¯}{n}}_{0},$$

$$\frac{\partial }{\partial C^{h}}\frac{1}{{\left(\lambda_{\text{f}}^{h}\right)}^{2}}=-\frac{1}{{\left(\lambda_{\text{f}}^{h}\right)}^{4}}f_{0}⊗f_{0},\;\frac{\partial }{\partial C^{h}}\frac{1}{{\left(\lambda_{\text{s}}^{h}\right)}^{2}}=-\frac{1}{{\left(\lambda_{\text{s}}^{h}\right)}^{4}}s_{0}⊗s_{0},\;\frac{\partial }{\partial C^{h}}\frac{1}{{\left(\lambda_{\text{n}}^{h}\right)}^{2}}=-\frac{1}{{\left(\lambda_{\text{n}}^{h}\right)}^{4}}{\overset{¯}{n}}_{0}⊗{\overset{¯}{n}}_{0},$$

$$\frac{\partial T_{\text{act}}^{h}}{\partial C^{h}}=\frac{T_{\text{ref}}}{r_{\text{s}}}\left(\frac{\overset{ˆ}{h}\left(\lambda_{\text{f}}^{h}\right)}{\partial C^{h}}\left[\left(\zeta_{\text{s}}+1\right)S+\zeta_{\text{w}}W\right]+\overset{ˆ}{h}\left(\lambda_{\text{f}}^{h}\right)\frac{\partial }{\partial C^{h}}\left[\left(\zeta_{\text{s}}+1\right)S+\zeta_{\text{w}}W\right]\right),$$

$$\frac{\partial \overset{ˆ}{h}\left(\lambda_{\text{f}}^{h}\right)}{\partial C^{h}}=\frac{1}{2}\frac{\partial \overset{ˆ}{h}\left(\lambda_{\text{f}}^{h}\right)}{\partial \lambda_{\text{f}}^{h}}\frac{1}{\lambda_{\text{f}}^{h}}f_{0}⊗f_{0},\;\frac{\partial \overset{ˆ}{h}\left(\lambda_{\text{f}}^{h}\right)}{\partial \lambda_{\text{f}}^{h}}=\left\{\begin{array}{cc} 0\;\; & \text{if}\;\lambda_{\text{f}}^{h}<\frac{1.87\beta_{0}-1}{2\beta_{0}}\\ 2\beta_{0}\; & \text{if}\;\frac{1.87\beta_{0}-1}{2\beta_{0}}\leq \lambda_{\text{f}}^{h}<0.87\\ \beta_{0}\; & \text{if}\;0.87\leq \lambda_{\text{f}}^{h}<1.2\\ 0\; & \text{if}\;\text{otherwise}, \end{array}\right)$$

 The rest of derivatives are dependent on the (Backward-Euler) time integration scheme used to solve q, such that

$$\frac{q_{n+1}-q_{n}}{\Delta t}=m_{q}\left(q_{n+1},c_{n+1},\lambda_{\text{f}\;n+1}^{h},\frac{\lambda_{\text{f}\;n+1}^{h}-\lambda_{\text{f}\;n}^{h}}{\Delta t}\right),$$

by first rearranging it into the residual form

$$R_{q}≔q_{n+1}-q_{n}-\Delta t\;m_{q}\left(q_{n+1},c_{n+1},\lambda_{\text{f}\;n+1}^{h},\frac{\lambda_{\text{f}\;n+1}^{h}-\lambda_{\text{f}\;n}^{h}}{\Delta t}\right)=0.$$

The derivatives of these residuals with respect to $\lambda_{\text{f}}^{h}$ are

$$\frac{\text{d}R_{q}}{\text{d}\lambda_{\text{f}}^{h}}=\frac{\partial R_{q}}{\partial \lambda_{\text{f}}^{h}}+\frac{\partial R_{q}}{\partial q}\frac{\partial q}{\partial \lambda_{\text{f}}^{h}}=0,$$

which, when rearranged, becomes

$$\frac{\partial q}{\partial \lambda_{\text{f}}^{h}}=-{\left(\frac{\partial R_{q}}{\partial q}\right)}^{-1}\frac{\partial R_{q}}{\partial \lambda_{\text{f}}^{h}},$$

where

$$\frac{\partial R_{q}}{\partial \lambda_{\text{f}}^{h}}=\left\{\begin{array}{c} 0\;\\ 0\;\\ \left\{\begin{array}{cc} 0\;\; & \text{if}\;\lambda_{\text{f}}^{h}\geq 1.2\\ \frac{\beta_{1}\;n_{\text{TRPN}}\Delta t\;k_{\text{TRPN}}\left(1-\text{CaTRPN}\right){\left[{\text{Ca}}^{2+}\right]}_{\text{int}}^{n_{\text{TRPN}}}}{{\left({\left[{\text{Ca}}^{2+}\right]}_{\text{T50}}^{\text{ref}}+\beta_{1}\left[\lambda_{\text{f}}^{h}+1\right]\right)}^{\left(n_{\text{TRPN}}-1\right)}}\;\; & \text{otherwise} \end{array}\right)\;\\ 0\;\\ -A_{\text{s}}\;\\ -A_{\text{w}},\; \end{array}\right);\;\frac{\partial R_{q}}{\partial q}=I_{6}-\Delta t\frac{\partial m_{q}}{\partial q}.$$

This leads to

$$\frac{\partial }{\partial C^{h}}\left[\left(\zeta_{\text{s}}+1\right)S+\zeta_{\text{w}}W\right]=\frac{\partial \zeta_{\text{s}}}{\partial \lambda_{\text{f}}^{h}}\frac{\partial \lambda_{\text{f}}^{h}}{\partial C^{h}}S+\frac{\partial \zeta_{\text{w}}}{\partial \lambda_{\text{f}}^{h}}\frac{\partial \lambda_{\text{f}}^{h}}{\partial C^{h}}W,$$

$$\frac{\partial \zeta_{\text{s}}}{\partial \lambda_{\text{f}}^{h}}\frac{\partial \lambda_{\text{f}}^{h}}{\partial C^{h}}=\frac{A_{\text{s}}}{2\lambda_{\text{f}}^{h}}f_{0}⊗f_{0},\;\frac{\partial \zeta_{\text{w}}}{\partial \lambda_{\text{f}}^{h}}\frac{\partial \lambda_{\text{f}}^{h}}{\partial C^{h}}=\frac{A_{\text{w}}}{2\lambda_{\text{f}}^{h}}f_{0}⊗f_{0}.$$

 Consequently, the final expression of the tangent modulus for the active part of the Second Piola–Kirchhoff stress reads as

$${\mathbb{C}}_{\text{act}}^{h}=2\frac{\partial S_{\text{act}}^{h}}{\partial C^{h}}=2[\frac{\partial \left(T_{\text{act}}^{h}+\sigma_{0}^{h}\right)}{\partial C^{h}}\frac{f_{0}⊗f_{0}}{{\left(\lambda_{\text{f}}^{h}\right)}^{2}}+\left(T_{\text{act}}^{h}+\sigma_{0}^{h}\right)\frac{\partial }{\partial C^{h}}\left(\frac{1}{{\left(\lambda_{\text{f}}^{h}\right)}^{2}}\right)f_{0}⊗f_{0}+\;k_{\text{ort}\;1}\frac{\partial T_{\text{act}}^{h}}{\partial C^{h}}\frac{s_{0}⊗s_{0}}{{\left(\lambda_{\text{s}}^{h}\right)}^{2}}+k_{\text{ort}\;1}T_{\text{act}}^{h}\frac{\partial }{\partial C^{h}}\left(\frac{1}{{\left(\lambda_{\text{s}}^{h}\right)}^{2}}\right)s_{0}⊗s_{0}+\;k_{\text{ort}\;1}\frac{\partial T_{\text{act}}^{h}}{\partial C^{h}}\frac{{\overset{¯}{n}}_{0}⊗{\overset{¯}{n}}_{0}}{{\left(\lambda_{\text{n}}^{h}\right)}^{2}}+k_{\text{ort}\;1}T_{\text{act}}^{h}\frac{\partial }{\partial C^{h}}\left(\frac{1}{{\left(\lambda_{\text{n}}^{h}\right)}^{2}}\right){\overset{¯}{n}}_{0}⊗{\overset{¯}{n}}_{0}]\approx \frac{1}{{\left(\lambda_{\text{f}}^{h}\right)}^{3}}\left[\frac{T_{\text{ref}}}{r_{\text{s}}}\left(\frac{\partial h\left(\lambda_{\text{f}}^{h}\right)}{\partial \lambda_{\text{f}}^{h}}\left[\zeta_{\text{s}}S+\zeta_{\text{w}}W\right]+h\left(\lambda_{\text{f}}^{h}\right)\left[\left(\frac{\partial \zeta_{\text{s}}}{\partial \lambda_{\text{f}}^{h}}S+\zeta_{\text{s}}\frac{\partial S}{\partial \lambda_{\text{f}}^{h}}\right)+\;\left(\frac{\partial \zeta_{\text{w}}}{\partial \lambda_{\text{f}}^{h}}S+\zeta_{\text{s}}\frac{\partial S}{\partial \lambda_{\text{f}}^{h}}\right)\right]\right)-2\frac{T_{\text{act}}^{h}+\sigma_{0}^{h}}{{\left(\lambda_{\text{f}}^{h}\right)}^{4}}\right]f_{0}⊗f_{0}⊗f_{0}⊗f_{0}+\;k_{\text{ort}\;1}\left[\frac{T_{\text{ref}}}{\lambda_{\text{f}}^{h}{\left(\lambda_{\text{s}}^{h}\right)}^{2}r_{\text{s}}}\left(\frac{\partial h\left(\lambda_{\text{f}}^{h}\right)}{\partial \lambda_{\text{f}}^{h}}\left[\zeta_{\text{s}}S+\zeta_{\text{w}}W\right]+h\left(\lambda_{\text{f}}^{h}\right)\left[\left(\frac{\partial \zeta_{\text{s}}}{\partial \lambda_{\text{f}}^{h}}S+\zeta_{\text{s}}\frac{\partial S}{\partial \lambda_{\text{f}}^{h}}\right)+\;\left(\frac{\partial \zeta_{\text{w}}}{\partial \lambda_{\text{f}}^{h}}S+\zeta_{\text{s}}\frac{\partial S}{\partial \lambda_{\text{f}}^{h}}\right)\right]\right)s_{0}⊗s_{0}⊗f_{0}⊗f_{0}-2\frac{T_{\text{act}}^{h}}{{\left(\lambda_{\text{s}}^{h}\right)}^{4}}s_{0}⊗s_{0}⊗s_{0}⊗s_{0}\right]+\;k_{\text{ort}\;2}\left[\frac{T_{\text{ref}}}{\lambda_{\text{f}}^{h}{\left(\lambda_{\text{n}}^{h}\right)}^{2}r_{\text{s}}}\left(\frac{\partial h\left(\lambda_{\text{f}}^{h}\right)}{\partial \lambda_{\text{f}}^{h}}\left[\zeta_{\text{s}}S+\zeta_{\text{w}}W\right]+h\left(\lambda_{\text{f}}^{h}\right)\left[\left(\frac{\partial \zeta_{\text{s}}}{\partial \lambda_{\text{f}}^{h}}S+\zeta_{\text{s}}\frac{\partial S}{\partial \lambda_{\text{f}}^{h}}\right)+\;\left(\frac{\partial \zeta_{\text{w}}}{\partial \lambda_{\text{f}}^{h}}S+\zeta_{\text{s}}\frac{\partial S}{\partial \lambda_{\text{f}}^{h}}\right)\right]\right){\overset{¯}{n}}_{0}⊗{\overset{¯}{n}}_{0}⊗f_{0}⊗f_{0}-2\frac{T_{\text{act}}^{h}}{{\left(\lambda_{\text{n}}^{h}\right)}^{4}}{\overset{¯}{n}}_{0}⊗{\overset{¯}{n}}_{0}⊗{\overset{¯}{n}}_{0}⊗{\overset{¯}{n}}_{0}\right].$$

In the previous relation, the approximation appears since $\frac{\partial \sigma_{0}^{h}}{\partial C^{h}}=0$; which in turn occurs because $\sigma_{0}$ only changes during the initiation phase, and it does so very smoothly.

## References

[1] World Health Organization (2018). The top 10 causes of death. http://www.who.int/news-room/fact-sheets/detail/the-top-10-causes-of-death.

[2] Rodriguez B., Carusi A., Abi-Gerges N., Ariga R., Britton O., Bub G., Bueno-Orovio A., Burton R.A., Carapella V., Cardone-Noott L., Daniels M.J., Davies M.R., Dutta S., Ghetti A., Grau V., Harmer S., Kopljar I., Lambiase P., Lu H.R., Lyon A., Mincholé A., Muszkiewicz A., Oster J., Paci M., Passini E., Severi S., Taggart P., Tinker A., Valentin J.-P., Varro A., Wallman M., Zhou X. (2015). Human-based approaches to pharmacology and cardiology: an interdisciplinary and intersectorial workshop. EP Eur..

[3] Niederer S.A., Lumens J., Trayanova N.A. (2019). Computational models in cardiology. Nat. Rev. Cardiol..

[4] Zemzemi N., Bernabeu M.O., Saiz J., Cooper J., Pathmanathan P., Mirams G.R., Pitt-Francis J., Rodriguez B. (2013). Computational assessment of drug-induced effects on the electrocardiogram: from ion channel to body surface potentials. Br. J. Pharmacol..

[5] Dutta S., Mincholé A., Zacur E., Quinn T.A., Taggart P., Rodriguez B. (2016). Early afterdepolarizations promote transmural reentry in ischemic human ventricles with reduced repolarization reserve. Prog. Biophys. Mol. Biol..

[6] O’Hara T., Virág L., Varró A., Rudy Y. (2011). Simulation of the undiseased human cardiac ventricular action potential: Model formulation and experimental validation. PLoS Comput. Biol..

[7] Strauss D.G., Gintant G., Li Z., Wu W., Blinova K., Vicente J., Turner J.R., Sager P.T. (2019). Comprehensive in vitro proarrhythmia assay (CiPA) update from a cardiac safety research consortium / health and environmental sciences institute / FDA meeting. Therapeutic Innovation & Regulatory Science.

[8] Passini E., Britton O.J., Lu H.R., Rohrbacher J., Hermans A.N., Gallacher D.J., Greig R.J.H., Bueno-Orovio A., Rodriguez B. (2017). Human in silico drug trials demonstrate higher accuracy than animal models in predicting clinical pro-arrhythmic cardiotoxicity. Front. Physiol..

[9] Land S., Park-Holohan S.-J., Smith N.P., dos Remedios C.G., Kentish J.C., Niederer S.A. (2017). A model of cardiac contraction based on novel measurements of tension development in human cardiomyocytes. J. Mol. Cell. Cardiol..

[10] Augustin C.M., Neic A., Liebmann M., Prassl A.J., Niederer S.A., Haase G., Plank G. (2016). Anatomically accurate high resolution modeling of human whole heart electromechanics: A strongly scalable algebraic multigrid solver method for nonlinear deformation. J. Comput. Phys..

[11] Favino M., Pozzi S., Pezzuto S., Prinzen F.W., Auricchio A., Krause R. (2016). Impact of mechanical deformation on pseudo-ECG: a simulation study. EP Eur..

[12] Franzone P.C., Pavarino L., Scacchi S. (2016). Joint influence of transmural heterogeneities and wall deformation on cardiac bioelectrical activity: A simulation study. Math. Biosci..

[13] Quarteroni A., Lassila T., Rossi S., Ruiz-Baier R. (2017). Integrated Heart–Coupling multiscale and multiphysics models for the simulation of the cardiac function. Comput. Methods Appl. Mech. Engrg..

[14] Santiago A., Aguado-Sierra J., Zavala-Aké M., Doste-Beltran R., Gómez S., Arís R., Cajas J.C., Casoni E., Vázquez M. (2018). Fully coupled fluid-electro-mechanical model of the human heart for supercomputers. Int. J. Numer. Methods Biomed. Eng..

[15] Dusturia N., Choi S.W., Song K.S., Lim K.M. (2019). Effect of myocardial heterogeneity on ventricular electro-mechanical responses: a computational study. Biomed. Eng. Online.

[16] Sugiura S., Washio T., Hatano A., Okada J., Watanabe H., Hisada T. (2012). Multi-scale simulations of cardiac electrophysiology and mechanics using the University of Tokyo heart simulator. Prog. Biophys. Mol. Biol..

[17] Arís Sánchez R. (2014). Electromechanical Large Scale Computational Models of the Ventricular Myocardium.

[18] Lafortune P., Arís R., Vázquez M., Houzeaux G. (2012). Coupled electromechanical model of the heart: Parallel finite element formulation. Int. J. Numer. Methods Biomed. Eng..

[19] Vázquez M., Houzeaux G., Koric S., Artigues A., Aguado-Sierra J., Arís R., Mira D., Calmet H., Cucchietti F., Owen H., Taha A., Burness E.D., Cela J.M., Valero M. (2016). Alya: Multiphysics engineering simulation toward exascale. J. Comput. Sci..

[20] Santiago A. (2018). Fluid-Electro-Mechanical Model of the Human Heart for Supercomputers.

[21] Dutta S., Mincholé A., Quinn T.A., Rodriguez B. (2017). Electrophysiological properties of computational human ventricular cell action potential models under acute ischemic conditions. Prog. Biophys. Mol. Biol..

[22] Costabal F.S., Concha F.A., Hurtado D.E., Kuhl E. (2017). The importance of mechano-electrical feedback and inertia in cardiac electromechanics. Comput. Methods Appl. Mech. Engrg..

[23] Barbarotta L., Rossi S., Dedé L., Quarteroni A. (2018). A transmurally heterogeneous orthotropic activation model for ventricular contraction and its numerical validation. International Journal for Numerical Methods in Biomedical Engineering.

[24] Fritz T., Wieners C., Seemann G., Steen H., Dössel O. (2014). Simulation of the contraction of the ventricles in a human heart model including atria and pericardium. Biomech. Model. Mechanobiol..

[25] Pezzuto S., Ambrosi D., Quarteroni A. (2014). An orthotropic active–strain model for the myocardium mechanics and its numerical approximation. Eur. J. Mech. A Solids.

[26] Pathmanathan P., Chapman S.J., Gavaghan D.J., Whiteley J.P. (2010). Cardiac electromechanics: The effect of contraction model on the mathematical problem and accuracy of the numerical scheme. Quart. J. Mech. Appl. Math..

[27] Eriksson T., Prassl A., Plank G., Holzapfel G. (2013). Influence of myocardial fiber/sheet orientations on left ventricular mechanical contraction. Math. Mech. Solids.

[28] Berberoǧlu E., Solmaz H.O., Gktepe S. (2014). Computational modeling of coupled cardiac electromechanics incorporating cardiac dysfunctions. Eur. J. Mech. A Solids.

[29] Hurtado D.E., Castro S., Madrid P. (2017). Uncertainty quantification of 2 models of cardiac electromechanics. Int. J. Numer. Methods Biomed. Eng..

[30] Zhou X., Bueno-Orovio A., Schilling R.J., Kirkby C., Denning C., Rajamohan D., Burrage K., Tinker A., Rodriguez B., Harmer S.C. (2019). Investigating the complex arrhythmic phenotype caused by the gain-of-function mutation KCNQ1-G229D. Front. Physiol..

[31] Levrero-Florencio F., Pankaj P. (2018). Using nonlinear homogenisation to improve the performance of macroscopic damage models of trabecular bone. Front. Physiol..

[32] Land S., Gurev V., Arens S., Augustin C.M., Baron L., Blake R., Bradley C., Castro S., Crozier A., Favino M., Fastl T.E., Fritz T., Gao H., Gizzi A., Griffith B.E., Hurtado D.E., Krause R., Luo X., Nash M.P., Pezzuto S., Plank G., Rossi S., Ruprecht D., Seemann G., Smith N.P., Sundnes J., Rice J.J., Trayanova N., Wang D., Jenny Wang Z., Niederer S.A. (2015). Verification of cardiac mechanics software: benchmark problems and solutions for testing active and passive material behaviour. Proc. R. Soc. A.

[33] Petersen S.E., Aung N., Sanghvi M.M., Zemrak F., Fung K., Paiva J.M., Francis J.M., Khanji M.Y., Lukaschuk E., Lee A.M., Carapella V., Kim Y.J., Leeson P., Piechnik S.K., Neubauer S. (2017). Reference ranges for cardiac structure and function using cardiovascular magnetic resonance (CMR) in caucasians from the UK Biobank population cohort. J. Cardiovasc. Magn. Reson..

[34] Holzapfel G.A., Ogden R.W. (2009). Constitutive modelling of passive myocardium: a structurally based framework for material characterization. Phil. Trans. R. Soc. A.

[35] Yin F.C., Chan C.C., Judd R.M. (1996). Compressibility of perfused passive myocardium. Am. J. Physiol. Heart Circ. Physiol..

[36] Moore C.C., McVeigh E.R., Zerhouni E.A. (1999). Noninvasive measurement of three-dimensional myocardial deformation with tagged magnetic resonance imaging during graded local ischemia. J. Cardiovasc. Magn. Reson..

[37] Leon L.J., Horáček B.M. (1991). Computer model of excitation and recovery in the anisotropic myocardium: I. Rectangular and cubic arrays of excitable elements. J. Electrocardiol..

[38] Rice J.J., Wang F., Bers D.M., de Tombe P.P. (2008). Approximate model of cooperative activation and crossbridge cycling in cardiac muscle using ordinary differential equations. Biophys. J..

[39] Usyk T., Mazhari R., McCulloch A. (2000). Effect of laminar orthotropic myofiber architecture on regional stress and strain in the canine left ventricle. J. Elasticity.

[40] Dorri F., Niederer P., Lunkenheimer P. (2006). A finite element model of the human left ventricular systole. Comput. Methods Biomech. Biomed. Eng..

[41] Propp A., Gizzi A., Levrero-Florencio F., Ruiz-Baier R. (2019). An orthotropic electro-viscoelastic model for the heart with stress-assisted diffusion. Biomechanics and Modeling in Mechanobiology.

[42] Nobile F., Quarteroni A., Ruiz Baier R. (2012). An active strain electromechanical model for cardiac tissue. Int. J. Numer. Methods Biomed. Eng..

[43] Trayanova N., Li W., Eason J., Kohl P. (2004). Effect of stretch-activated channels on defibrillation efficacy. Heart Rhythm.

[44] Sachs F. (2010). Stretch-activated ion channels: What are they?. Physiology.

[45] Ruiz-Baier R. (2015). Primal-mixed formulations for reaction-diffusion systems on deforming domains. J. Comput. Phys..

[46] Colli Franzone P., Pavarino L.F., Scacchi S. (2016). Bioelectrical effects of mechanical feedbacks in a strongly coupled cardiac electro-mechanical model. Math. Models Methods Appl. Sci..

[47] Lopez-Yunta M. (2018). Multimodal Ventricular Tachycardia Analysis: Towards the Accurate Parametrization of Predictive HPC Electrophysiological Computational Models.

[48] Karypis G., Kumar V. (1998). A fast and high quality multilevel scheme for partitioning irregular graphs. SIAM J. Sci. Comput..

[49] Clayton R., Panfilov A. (2008). A guide to modelling cardiac electrical activity in anatomically detailed ventricles. Prog. Biophys. Mol. Biol..

[50] Streeter D.D., Spotnitz H.M., Patel D.P., Ross J., Sonnenblick E.H. (1969). Fiber orientation in the canine left ventricle during diastole and systole. Circ. Res..

[51] McKay M.D., Beckman R.J., Conover W.J. (1979). A comparison of three methods for selecting values of input variables in the analysis of output from a computer code. Technometrics.

[52] Helton J.C., Davis F.J. (2002). Illustration of sampling-based methods for uncertainty and sensitivity analysis. Risk Anal..

[53] Marino S., Hogue I.B., Ray C.J., Kirschner D.E. (2008). A methodology for performing global uncertainty and sensitivity analysis in systems biology. J. Theoret. Biol..

[54] Helton J., Johnson J., Sallaberry C., Storlie C. (2006). Survey of sampling-based methods for uncertainty and sensitivity analysis. Reliab. Eng. Syst. Saf..

[55] National Academy of Sciences J. (2012). Assessing the Reliability of Complex Models: Mathematical and Statistical Foundations of Verification, Validation and Uncertainty Quantification.

[56] Britton O.J., Bueno-Orovio A., Van Ammel K., Lu H.R., Towart R., Gallacher D.J., Rodriguez B. (2013). Experimentally calibrated population of models predicts and explains intersubject variability in cardiac cellular electrophysiology. Proc. Natl. Acad. Sci..

[57] Muszkiewicz A., Liu X., Bueno-Orovio A., Lawson B.A.J., Burrage K., Casadei B., Rodriguez B. (2018). From ionic to cellular variability in human atrial myocytes: an integrative computational and experimental study. Am. J. Physiol. Heart Circ. Physiol..

[58] Pueyo E., Dangerfield C.E., Britton O.J., Virág L., Kistamás K., Szentandrássy N., Jost N., Varró A., Nánási P.P., Burrage K., Rodríguez B. (2018). Correction: Experimentally-based computational investigation into beat-to-beat variability in ventricular repolarization and its response to ionic current inhibition. PLoS One.

[59] Iman R.L., Conover W.J. (1979). The use of the rank transform in regression. Technometrics.

[60] Saltelli A., Sobol’ I.M. (1995). About the use of rank transformation in sensitivity analysis of model output. Reliab. Eng. Syst. Saf..

[61] Iman R.L., Helton J.C. (1991). The repeatability of uncertainty and sensitivity analyses for complex probabilistic risk assessments. Risk Anal..

[62] Helton J., Johnson J., McKay M., Shiver A., Sprung J. (1995). Robustness of an uncertainty and sensitivity analysis of early exposure results with the MACCS reactor accident consequence model. Reliab. Eng. Syst. Saf..

[63] Dusch M.N., Thadani S.R., Dhillon G.S., Hope M.D. (2014). Diastolic function assessed by cardiac MRI using longitudinal left ventricular fractional shortening. Clin. Imaging.

[64] Dong S.J., MacGregor J.H., Crawley A.P., McVeigh E.R., Belenkie I., Smith E.R., Tyberg J.V., Beyar R. (1994). Left ventricular wall thickness and regional systolic function in patients with hypertrophic cardiomyopathy. A three-dimensional tagged magnetic resonance imaging study. Circulation.

[65] Alter P., Rupp H., Rominger M., Vollrath A., Czerny F., Klose K., Maisch B. (2007). Relation of B-type natriuretic peptide to left ventricular wall stress as assessed by cardiac magnetic resonance imaging in patients with dilated cardiomyopathy. Can. J. Physiol. Pharmacol..

[66] Emilsson K., Egerlid R., Nygren B.-M., Wandt B. (2006). Mitral annulus motion versus long-axis fractional shortening. Exp. Clin. Cardiol..

[67] Sechtem U., Sommerhoff B.A., Markiewicz W., White R.D., Cheitlin M.D., Higgins C.B. (1987). Regional left ventricular wall thickening by magnetic resonance imaging: Evaluation in normal persons and patients with global and regional dysfunction. Amer. J. Cardiol..

[68] Zhang Y., Guallar E., Blasco-Colmenares E., Butcher B., Norgard S., Nauffal V., Marine J.E., Eldadah Z., Dickfeld T., Ellenbogen K.A., Tomaselli G.F., Cheng A. (2015). Changes in follow-up left ventricular ejection fraction associated with outcomes in primary prevention implantable cardioverter-defibrillator and cardiac resynchronization therapy device recipients. J. Am. Coll. Cardiol..

[69] Beyar R., Shapiro E.P., Graves W.L., Rogers W.J., Guier W.H., Carey G.A., Soulen R.L., Zerhouni E.A., Weisfeldt M.L., Weiss J.L. (1990). Quantification and validation of left ventricular wall thickening by a three-dimensional volume element magnetic resonance imaging approach. Circulation.

[70] Lima J.A., Jeremy R., Guier W., Bouton S., Zerhouni E.A., McVeigh E., Buchalter M.B., Weisfeldt M.L., Shapiro E.P., Weiss J.L. (1993). Accurate systolic wall thickening by nuclear magnetic resonance imaging with tissue tagging: Correlation with sonomicrometers in normal and ischemic myocardium. J. Am. Coll. Cardiol..

[71] De Larochellière A., Le Ven F., Tizón-Marcos H., Bibeau K., Pibarot P., Deneault-Bissonnette S., Larose A., Deschepper C.F. (2015). Cardiac morphology and function reference values derived from a large subset of healthy young caucasian adults by magnetic resonance imaging. Eur. Heart J. Cardiovasc. Imaging.

[72] Wang Z.J., Wang V.Y., Bradley C.P., Nash M.P., Young A.A., Cao J.J. (2018). Left ventricular diastolic myocardial stiffness and end-diastolic myofibre stress in human heart failure using personalised biomechanical analysis. J. Cardiovasc. Transl. Res..

[73] Kurita A., Itoh H., Sato F., Ichibori Y., Yoshida A. (2008). Longitudinal fractional shortening and its relation to diastolic cardiac function. J. Med. Ultrasonics.

[74] de Simone G., Ganau A., Roman M., Devereux R. (1997). Relation of left ventricular longitudinal and circumferential shortening to ejection fraction in the presence or in the absence of mild hypertension. J. Hypertens..

[75] Pflugfelder P.W., Sechtem U.P., White R.D., Higgins C.B. (1988). Quantification of regional myocardial function by rapid cine MR imaging. Am. J. Roentgenol..

[76] Hsu J.J., Ziaeian B., Fonarow G.C. (2017). Heart failure with mid-range (borderline) ejection fraction. JACC Heart Fail..

[77] Coppini R., Ferrantini C., Yao L., Fan P., Del Lungo M., Stillitano F., Sartiani L., Tosi B., Suffredini S., Tesi C., Yacoub M., Olivotto I., Belardinelli L., Poggesi C., Cerbai E., Mugelli A. (2013). Late sodium current inhibition reverses electromechanical dysfunction in human hypertrophic cardiomyopathyclinical perspective. Circulation.

[78] ten Tusscher K.H.W.J., Noble D., Noble P.J., Panfilov A.V. (2004). A model for human ventricular tissue. Am. J. Physiol. Heart Circ. Physiol..

[79] Rossi S., Lassila T., Ruiz-Baier R., Sequeira A., Quarteroni A. (2014). Thermodynamically consistent orthotropic activation model capturing ventricular systolic wall thickening in cardiac electromechanics. Eur. J. Mech. A.

[80] Cheng A., Nguyen T.C., Malinowski M., Daughters G.T., Miller D.C., Ingels N.B. (2008). Heterogeneity of left ventricular wall thickening mechanisms. Circulation.

[81] Nielles-Vallespin S., Khalique Z., Ferreira P.F., de Silva R., Scott A.D., Kilner P., McGill L.-A., Giannakidis A., Gatehouse P.D., Ennis D., Aliotta E., Al-Khalil M., Kellman P., Mazilu D., Balaban R.S., Firmin D.N., Arai A.E., Pennell D.J. (2017). Assessment of myocardial microstructural dynamics by in vivo diffusion tensor cardiac magnetic resonance. J. Am. Coll. Cardiol..

[82] Kawel N., Turkbey E.B., Carr J.J., Eng J., Gomes A.S., Hundley W.G., Johnson C., Masri S.C., Prince M.R., van der Geest R.J., Lima J.A., Bluemke D.A. (2012). Normal left ventricular myocardial thickness for middle-aged and older subjects with steady-state free precession cardiac magnetic resonance. Circ. Cardiovasc. Imaging.

[83] Ambrosi D., Pezzuto S. (2012). Active stress vs. Active strain in mechanobiology: Constitutive issues. J. Elasticity.

[84] Nolan D., Gower A., Destrade M., Ogden R., McGarry J. (2014). A robust anisotropic hyperelastic formulation for the modelling of soft tissue. J. Mech. Behav. Biomed. Mater..

[85] McEvoy E., Holzapfel G.A., McGarry P. (2018). Compressibility and anisotropy of the ventricular myocardium: Experimental analysis and microstructural modeling. J. Biomech. Eng..

[86] Oliveira B.L.d., Pfeiffer E.R., Sundnes J., Wall S.T., McCulloch A.D. (2015). Increased cell membrane capacitance is the dominant mechanism of stretch-dependent conduction slowing in the rabbit heart: A computational study. Cell. Mol. Bioeng..

[87] Mills R.W., Narayan S.M., McCulloch A.D. (2008). Mechanisms of conduction slowing during myocardial stretch by ventricular volume loading in the rabbit. Am. J. Physiol. Heart Circ. Physiol..

[88] Pueyo E., Orini M., Rodríguez J.F., Taggart P. (2016). Interactive effect of beta-adrenergic stimulation and mechanical stretch on low-frequency oscillations of ventricular action potential duration in humans. J. Mol. Cell. Cardiol..

[89] Hurtado D.E., Kuhl E. (2014). Computational modelling of electrocardiograms: repolarisation and T-wave polarity in the human heart. Comput. Methods Biomech. Biomed. Eng..

[90] Niederer S.A., Smith N.P. (2008). An improved numerical method for strong coupling of excitation and contraction models in the heart. Prog. Biophys. Mol. Biol..

